# Multi-ethnic genome-wide association analyses of white blood cell and platelet traits in the Population Architecture using Genomics and Epidemiology (PAGE) study

**DOI:** 10.1186/s12864-021-07745-5

**Published:** 2021-06-09

**Authors:** Yao Hu, Stephanie A. Bien, Katherine K. Nishimura, Jeffrey Haessler, Chani J. Hodonsky, Antoine R. Baldassari, Heather M. Highland, Zhe Wang, Michael Preuss, Colleen M. Sitlani, Genevieve L. Wojcik, Ran Tao, Mariaelisa Graff, Laura M. Huckins, Quan Sun, Ming-Huei Chen, Abdou Mousas, Paul L. Auer, Guillaume Lettre, the Blood Cell Consortium, Weihong Tang, Lihong Qi, Bharat Thyagarajan, Steve Buyske, Myriam Fornage, Lucia A. Hindorff, Yun Li, Danyu Lin, Alexander P. Reiner, Kari E. North, Ruth J. F. Loos, Laura M. Raffield, Ulrike Peters, Christy L. Avery, Charles Kooperberg

**Affiliations:** 1 Public Health Sciences Division, Fred Hutchinson Cancer Research Center, Seattle, WA USA; 2 Department of Epidemiology, Gillings School of Public Health, University of North Carolina at Chapel Hill, Chapel Hill, NC USA; 3 The Charles Bronfman Institute for Personalized Medicine, Icahn School of Medicine at Mount Sinai, New York, NY USA; 4 Cardiovascular Health Research Unit, University of Washington, Seattle, WA USA; 5 Stanford University School of Medicine, Stanford, CA USA; 6 Department of Biostatistics, Vanderbilt University Medical Center, Nashville, TN USA; 7 The Vanderbilt Genetics Institute, Division of Genetic Medicine, Vanderbilt University Medical Center, Nashville, TN USA; 8 Pamela Sklar Division of Psychiatric Genomics, Icahn School of Medicine at Mount Sinai, New York, NY USA; 9 Department of Biostatistics, Gillings School of Public Health, University of North Carolina at Chapel Hill, Chapel Hill, NC USA; 10 The Framingham Heart Study, National Heart, Lung and Blood Institute, Framingham, MA USA; 11 Population Sciences Branch, Division of Intramural Research, National Heart, Lung and Blood Institute, Framingham, MA USA; 12 Montreal Heart Institute, Montreal, Quebec Canada; 13 School of Public Health, University of Wisconsin–Milwaukee, Milwaukee, WI USA; 14 Department of Medicine, Faculty of Medicine, Université de Montréal, Montreal, Quebec Canada; 15 School of Public Health, University of Minnesota, Minneapolis, MN USA; 16 School of Medicine, University of California Davis, Davis, CA USA; 17 Medical School of University of Minnesota, Minneapolis, MN USA; 18 Department of Statistics and Biostatistics, Rutgers University, Piscataway, NJ USA; 19 Brown Foundation Institute for Molecular Medicine, the University of Texas Health Science Center, Houston, TX USA; 20 Division of Genomic Medicine, NIH National Human Genome Research Institute, Bethesda, MD USA; 21 Department of Genetics, Gillings School of Public Health, University of North Carolina at Chapel Hill, Chapel Hill, NC USA

**Keywords:** GWAS, White blood cells, Platelets, Multi-ethnic

## Abstract

**Background:**

Circulating white blood cell and platelet traits are clinically linked to various disease outcomes and differ across individuals and ancestry groups. Genetic factors play an important role in determining these traits and many loci have been identified. However, most of these findings were identified in populations of European ancestry (EA), with African Americans (AA), Hispanics/Latinos (HL), and other races/ethnicities being severely underrepresented.

**Results:**

We performed ancestry-combined and ancestry-specific genome-wide association studies (GWAS) for white blood cell and platelet traits in the ancestrally diverse Population Architecture using Genomics and Epidemiology (PAGE) Study, including 16,201 AA, 21,347 HL, and 27,236 EA participants. We identified six novel findings at suggestive significance (*P* < 5E-8), which need confirmation, and independent signals at six previously established regions at genome-wide significance (*P* < 2E-9). We confirmed multiple previously reported genome-wide significant variants in the single variant association analysis and multiple genes using PrediXcan. Evaluation of loci reported from a Euro-centric GWAS indicated attenuation of effect estimates in AA and HL compared to EA populations.

**Conclusions:**

Our results highlighted the potential to identify ancestry-specific and ancestry-agnostic variants in participants with diverse backgrounds and advocate for continued efforts in improving inclusion of racially/ethnically diverse populations in genetic association studies for complex traits.

**Supplementary Information:**

The online version contains supplementary material available at 10.1186/s12864-021-07745-5.

## Background

White blood cell and platelet traits are important indicators of health status and can be used to diagnose and assess the risk of cardiovascular diseases [1–4], immunologic disorders [5, 6], and cancers [7, 8]. These traits, including white blood cell count (WBC), basophil (BAS), eosinophil (EOS), lymphocyte (LYM), monocyte (MON), neutrophil (NEU), platelet count (PLT) and mean platelet volume (MPV), are complex and heritable, with the estimated heritability ranging from 13.8% (BAS) to over 50% for PLT and MPV [9–11]. Many genetic variants have been identified through genome-wide association studies (GWAS) and exome-wide association analyses for white blood cell and platelet traits [11–22]. However, a gap between estimated heritability and proportion of variance explained by identified variants remains, suggesting that additional loci remain undiscovered [12].

In addition, the majority of large-scale white blood cell and platelet count genomic studies were conducted in populations of European ancestry (EA) despite differences in the genetic architecture of white blood cell and platelet traits across ancestral groups [23]. For example, populations of African ancestry have lower WBC and NEU levels compared to other race/ethnic groups [24] while Hispanic/Latino (HL) populations tend to have higher WBC and NEU levels compared to non-Hispanic white populations [25]. Ancestry-specific genetic variants also have been reported for white blood cell and platelet traits, including the Duffy/*DARC* null variant (rs2814778) associated with lower WBC and NEU in African populations [26, 27].

The Population Architecture using Genomics and Epidemiology (PAGE) Study funded by the National Human Genome Research Institute and the National Institute on Minority Health and Health Disparities was initiated to systematically characterize the genetic architecture underlying complex diseases and related quantitative traits among underrepresented minority populations in the U.S. through large-scale genetic epidemiological research [28]. We previously developed the Multiethnic Genotyping Array (MEGA) to improve variant coverage of the genome across multiple underrepresented populations [29]. By taking advantage of this tailored genotyping array, we performed ancestry-combined as well as ancestry-specific association analyses of the eight white blood cell and platelet traits in African American (AA), HL, and EA populations, aiming to identify novel genetic loci, dissect association signals at previously established regions, and strengthen understanding of the genetic architecture of white blood cell and platelet traits by improving diversity.

## Results

A maximum of 64,784 participants were included in discovery association analysis (Table 1 and Supplemental Table 1). Mean values for white blood cell and platelet traits varied by race/ethnicity, with the highest means of WBC, EOS, and MON observed in HL participants; the highest BAS, LYM, PLT, and MPV level in AA participants; and the highest NEU level in EA participants (Table 1). In the absence of evidence of genomic inflation in ancestry-specific and ancestry-combined meta-analysis (ranged: 0.936 to 1.150, Supplemental Table 2), the number of loci that reached genome-wide significance ([*P* < 2E-9, based on minor allele frequency (MAF)-specific *P*-value thresholds) [30] or suggestive significance (*P* < 5E-8) in the single-trait analyses combining all ancestries were 37 and 46 for WBC, 3 and 4 for BAS and EOS, 4 and 7 for LYM, 14 and 21 for MON and MPV, 29 and 35 for NEU, and 19 and 26 for PLT, respectively. The ancestry-combined analysis identified more genome-wide significant and suggestively significant loci compared to the ancestry-specific analysis for all phenotypes, except for WBC and NEU. The largest numbers of significant findings for WBC and NEU were from AA-specific analysis, most of which showed significant associations only in the AA (Supplemental Table 3). Top variants at significant loci in the ancestry-combined and ancestry-specific analysis are presented in Supplemental Table 3.

**Table 1:** Characteristics of the ancestrally diverse populations in PAGE

	AA	HL	EA
N ^a^	16,201	21,347	27,236
Age (years)	56.35(12.39)	51.29(14.24)	60.42(12.40)
Female (%)	84.46	69.05	79.95
Measurements ^b^			
WBC	5.76(1.93)	6.52(1.96)	6.16(1.70)
BAS ^c^	0.043(0.032)	0.039(0.033)	0.040(0.033)
EOS ^c^	0.16(0.12)	0.18(0.13)	0.16(0.13)
LYM	2.24(0.98)	2.15(0.82)	2.06(0.92)
MON	0.44(0.21)	0.52(0.18)	0.44(0.24)
NEU	3.19(1.56)	3.77(1.56)	3.82(1.37)
PLT	250.17(64.63)	245.70(64.58)	248.84(58.87)
MPV	9.89(1.71)	9.60(1.51)	9.70(1.75)

*AA* African American; *HL* Hispanic/Latino; *EA* European ancestry; *N* sample size; *WBC* white blood cell count; *BAS* basophil; *EOS* eosinophil; *LYM* lymphocyte; *MON*, monocyte; *NEU* neutrophil; *PLT* platelet count; *MPV* mean platelet volume

^a^ The largest sample size for all the traits was presented

^b^ Values are shown as mean (SD)

^c^ BAS and EOS levels were imputed for AA, HL, and EA participants, respectively

### Identification of novel loci and novel associations at established loci

In the discovery stage, we identified two novel loci that reached suggestive significance (*INSIG1* and *IGF1*, *P* < 5E-8, Table 2, Supplemental Fig. 1). The lead variant at *IGF1* is common (MAF > 20%) and the association signals for LYM were driven by all three ancestral groups (Table 2). The lead variant at *INSIG1*, however, showed evidence of association with BAS only in AA and HL populations (Table 2). In addition, we identified four novel associations at suggestive significance at previously established white blood cell and platelet loci, here identifying novel associations with WBC (*TG*), NEU (*MED13L*), and MPV (*HADHB* and *PPP1R16B*). Among these four novel associations at established loci, three showed ancestry specificity with the lead variants being monomorphic in at least one ancestral population (the lead variants at *MED13L* and *HADHB* are monomorphic in EA populations while the lead variant at *PPP1R16B* is monomorphic in AA and HL populations, Table 2). The six novel findings we identified in the single-trait association analysis also showed suggestive evidence of association in the multi-trait analysis (*P*≤9.85E-7, Supplemental Table 4).

**Table 2:** Novel findings identified in the ancestry-specific and ancestry-combined meta-analyses ^a^

Variant	Trait	Gene	Chr:Pos	CA/ NCA	CAF (%) (AA/HA/ EA)	AA-specific analysis		HL-specific analysis		EA-specific analysis		Ancestry-combined analysis	
						BETA	*P*	BETA	*P*	BETA	*P*	BETA	*P*
rs7832064	WBC	*TG*	8:133960430	T/C	45/26/18	0.05	2.16E-5	0.03	6.48E-3	0.03	4.98E-3	0.04	1.61E-8
**rs76582140**	**BAS**	***INSIG1***	**7:155017263**	**A/G**	**2.0/8.1/1.2**	**0.24**	**3.92E-4**	**0.10**	**6.72E-6**	**0.08**	**0.27**	**0.11**	**3.86E-8**
**rs59314616**	**LYM**	***IGF1***	**12:102955278**	**AT/A**	**31/21/23**	**−0.07**	**1.43E-4**	**−0.06**	**2.50E-4**	**−0.05**	**8.82E-3**	**−0.06**	**5.51E-9**
rs76307330	NEU	*MED13L*	12:115814083	A/G	0.6/0.1/0	0.57	1.14E-5	0.67	8.83E-4	–	–	0.60	4.09E-8
rs571446846	MPV	*HADHB*	2:26519245	AT/A	0.2/0.2/0	0.90	1.15E-3	1.12	8.00E-6	–	–	1.02	4.02E-8
rs567151067	MPV	*PPP1R16B*	20:37534937	G/T	0/0/0.1	–	–	–	–	−2.67	3.04E-8	−2.67	3.04E-8

*Chr* chromosome; *Pos* position; *CA* coded allele; *NCA* non-coded allele; *CAF* coded allele frequency; *AA* African American; *HL* Hispanic/Latino; *EA* European ancestry; *WBC* white blood cell; *BAS* basophil; *LYM* lymphocyte; *NEU* neutrophil; *MPV* mean platelet volume

^a^ Genome-wide significance and suggestive significance were defined as *P*<2E-9 and *P*<5E-8, respectively. Novel loci that have not been reported for any of the eight studied traits are presented in bold

In the replication stage, the two novel loci and the four novel associations at established loci were examined in independent populations from the Blood Cell Consortium (BCX) [23] after excluding the overlapped BioMe multi-ethnic and WHI EA samples. BCX represents the largest published trans-ethnic meta-analysis of blood cell traits, with a total of 746,667 participants (76% EA, 20% East Asian, 2% AA, 1% HL, and 1% South Asian). None of the six loci showed evidence of association in the replication stage (Supplemental Table 5). All variants showed consistent directions except for the *MED13L* variant.

### Identification of genes from the PrediXcan analysis

Next, we examined associations between genetically regulated gene expression (GREx) and WBC and PLT levels, identifying 207 significant genes that mapped to previously reported loci [false discovery rate (FDR)< 0.05, Supplemental Table 6]. Four out of eleven (*DARC*, *NRBP1*, *HLA*, and *MED24*) and nine out of eighteen (*PLOD1*, *LDLRAP1*, *TAPBP*, *BAK1*, *MAPK13*, *PLEC*, *TRAF3*, *ZFP14*, and *ZNF793*) regions were associated with WBC and PLT, respectively, and harbored more than one gene, which could indicate either multiple functional genes at these regions or co-regulation by variant predictors and correlation of expression levels of neighboring genes (Supplemental Table 6).

### Evaluation of previously reported loci

A literature review that included genetic variants indexed in the GWAS Catalog (latest access date: September 23, 2020) or targeted chips (Metabochip or Exomechip) [11–22, 31] identified a total of 8531 unique variants mapping to 1158 known white blood cell and platelet loci (based on 2 Mb genomic regions). After restricting to WBC, BAS, EOS, LYM, MON, NEU, PLT, and MPV as well as variants that passed quality control (QC) thresholds in PAGE, 7807 variants at 1099 loci were available. A total of 20 (WBC), 3 (BAS), 3 (EOS), 3 (LYM), 11 (MON), 19 (NEU), 17 (PLT), and 14 (MPV) previously identified loci reached genome-wide significance in the ancestry-combined meta-analysis (based on 2 Mb regions, *P*<2E-9). A total of 1953 variants at 148 loci showed significant evidence of association with at least one of the eight traits after Bonferroni correction (*P*< 4.32E-5, 0.05/1158) and 5526 variants at 805 loci had *P*< 0.05 in PAGE (Supplemental Table 7).

We then evaluated loci reported by the largest Euro-centric GWAS [12] at the time of analysis to compare effect sizes across EA, AA, and HL populations. Due to the relatively limited sample size of EA populations in PAGE (maximum sample size of 27,236), we used the summary statistics from the EA-specific meta-analysis in the BCX Consortium (maximum sample size of 563,946) for estimates of EA effect sizes [23]. A total of 1287 unique variants [164 (WBC), 72 (BAS), 184 (EOS), 159 (LYM), 220 (MON), 134 (NEU), 259 (PLT), and 268 (MPV)] were available in all three ancestral groups. Of the 16 comparisons (eight traits and two ancestral groups), 14 showed significant correlation (*P*< 3.13E-3, 0.05/16, Supplemental Table 8). Effect sizes were smaller in AA and HL compared to EA populations, with the exception of BAS-associated loci between HL and EA populations and LYM-associated loci between AA and EA populations (Supplemental Table 8, Supplemental Fig. 2). The highest phenotypic variance explained by these loci was observed in EA populations for seven of the eight traits (Supplemental Table 9). The largest variance explained for NEU was observed in AA populations, driven by the Duffy/*DARC* null variant (rs2814778, explaining over 11% variance). Since the paper that reported these loci was also a part of the BCX Consortium, we further calculated effect sizes in EA populations to account for the winner’s curse [32]. The results remained largely unchanged (Supplemental Tables 8 and 9).

To dissect association signals at previously established regions, we performed stepwise conditional analysis by adjusting for the most significant variant in each round. We identified genome-wide significant independent signals at six reported loci (*DARC* for WBC and NEU; *MED24* for WBC; and *BAK1*, *HBS1L*, *AK3*, and *SH2B3* for PLT, *P*<2E-9): each locus harbored two independent variants in PAGE (Supplemental Table 10). The independent variants at the *DARC* and *SH2B3* loci were mainly driven by signals in AA and/or HL populations while variants at the other four loci were jointly driven by signals across the three ancestral groups (Supplemental Table 10).

### Functional annotation

Bioinformatic follow-up of the two suggestive loci and four novel associations at established loci (Supplemental Fig. 3) identified putative transcription factors (rs116377097 and rs75640787 overlapped with KAP1 and EZH2, respectively, Supplemental Fig. 3B) for the *INSIG1* locus. At the *HADHB* locus, two variants in moderate linkage disequilibrium (LD) with the lead variant overlapped with enhancer and repressor activities (rs543901501, r^2^=0.47) and transcribed regions (rs77943157, r^2^=0.50), respectively (Supplemental Fig. 3E). At the *TG* locus, a total of 59 variants were associated with gene expression levels of *LRRC6* in whole blood [33] (Supplemental Table 11), and three variants showed DANN rank score > 0.9 (deleterious, Supplemental Table 11). We examined the six novel findings and their LD proxies in the gchromVAR results using the UK BioBank (UKBB) and BCX data, which quantified the enrichment of the 95% credible set variants from the trans-ethnic and ethnic-specific results within regions of accessible chromatin identified by ATAC-seq in 18 hematopoietic populations [34, 35]. However, none of our novel findings and their LD proxies showed posterior probability (PP)> 0.01 in UKBB EA populations or PP> 0.001 in BCX ancestrally diverse populations (data not shown).

Annotation of the independent variants we identified in the stepwise conditional analysis was performed by extracting information of each available variant from the whole genome sequence annotator (WGSA) dataset (Supplemental Table 12). Two variants at the *DARC* locus and one variant at the *SH2B3* locus showed a DANN rank score > 0.9 (deleterious, Supplemental Table 12). Two variants at the *AK3* locus and one variant each at the *SH2B3* and *MED24* loci showed Eigen PC phred scores ≥ 17 (functional, Supplemental Table 12). We also examined these independent variants in the BCX gchromVAR results. The most significant variant, the well-established functional variant rs2814778 at the *DARC* locus [27], showed PP=1 for multiple white blood cell related traits in the fine-mapping results based on the BCX trans-ethnic results as well as AA- and HL-specific results (Supplemental Table 13).

## Discussion

We performed GWAS meta-analyses of white blood cell and platelet traits in ancestrally diverse populations from the PAGE Study and identified two novel loci and added novel associations to four loci previously linked to white blood cell and platelet traits. We also observed independent signals at six previously reported loci at genome-wide significance, two of which were mainly driven by signals in AA and/or HL populations. Evaluation in PAGE of loci previously reported by a Euro-centric GWAS indicated attenuation of effect estimates in AA and HL compared to EA populations even after accounting for the winner’s curse.

The two novel loci, *INSIG1* (associated with BAS) and *IGF1* (associated LYM), have not been linked to any white blood cell or platelet traits before, although we acknowledge lack of replication. These two genes (*INSIG1*, insulin induced gene 1; *IGF1*, insulin like growth factor 1) are both closely related to glucose and lipids homeostasis, which have a complex interplay with inflammation and related traits. The lead variant and its LD proxies at the *INSIG1* locus are located in an intergenic region close to another gene *HTR5A* (5-hydroxytryptamine receptor 5A), which has been reported for sulfasalazine-induced agranulocytosis in EA populations [36]. Two LD proxies of the lead variants overlapped with transcription factors KAP1 and EZH2. KAP1 (KRAB Associated Protein 1), also known as tripartite motif-containing 28 (TRIM28), has been reported as an essential factor in the erythroblast differentiation in a mouse model [37] while EZH2 (enhancer of Zeste homolog 2) plays an important role in T cell differentiation and function [38]. Future studies are needed to better understand the connection between these two novel loci and the associated traits. At the *TG* locus which was reported for PLT and EOS and was associated with WBC in PAGE, the lead variant and multiple LD proxies are intronic variants of the *TG* (thyroglobulin) gene and also exhibited association with gene expression level of *LRRC6* (leucine rich repeat containing 6) in whole blood (Supplemental Table 11). Evidence from gene expression data in the Consortium for the Architecture of Gene Expression (CAGE), the Depression Genes and Networks (DGN), the eQTLGen Consortium, and the Netherlands Study of Depression and Anxiety/the Netherlands Twin Register (NESDA/NTR) all supported it as a cis-eQTL of *LRRC6* (*P*≤1.3E-17, data not shown). Polymorphisms in *TG* gene are associated with susceptibility to autoimmune thyroid diseases (AITD) while defects in *LRRC6* gene are a cause of primary ciliary dyskinesia-19, which features chronic infections and persistent inflammation of the respiratory system [39]. Functional studies are needed to identify the functional gene(s) at this locus. Nevertheless, all these novel findings need to be confirmed in independent studies. The failure to replicate our novel findings in the BCX Consortium may have, at least in part, resulted from the relatively modest sample sizes of AA and HL samples in BCX (roughly 13,000 AA and 6500 HL participants after excluding overlapping BioMe multi-ethnic samples), as most of our novel findings are more common in AA and HL populations. The one variant whose association was found in EA (*PPP1R16B-*rs567151067) is available only in one of the participating studies of European ancestry. This may be because this variant is of low frequency in Europeans (MAF=0.001 in PAGE European populations and MAF=0.0004 in BCX Consortium).

Our findings highlighted the potential of uncovering additional genetic loci in ancestrally diverse populations, especially those showing ancestry-specificity in underrepresented populations and were possibly missed by previous Euro-centric analyses. Among the six novel findings we identified in the ancestry-combined meta-analysis, half of them were mainly driven by association signals in AA and HL populations. Among these three loci, two of them harbored lead variants that were monomorphic in EA populations (*MED13L* and *HADHB*) and the lead variant at *INSIG1* exhibited relatively lower MAF in EA. Among the six loci showing evidence of independent signals in the stepwise conditional analysis, two of them harbored variants whose associations were mainly driven by AA and/or HL populations. One is the *DARC* locus, where the two variants associated with WBC or NEU were independent of the well-established functional variant rs2814778 [27] and one of the variants, rs13375519, showed a DANN rank score > 0.9 (deleterious). The other one is the *SH2B3* locus, where the most significant variant, rs3742003, was associated with expression levels of multiple genes in various tissues and showed a DANN rank score > 0.9 (deleterious) and an Eigen PC phred score ≥17 (functional). These annotation findings indicated potential functions of these independent variants at the two established regions and merit further investigation. More significant loci were identified in the ancestry-combined analysis except for WBC and NEU, where most significant results were in AA, suggesting improved power when combining all samples and the potential to uncover loci driven by all ancestral groups.

The ancestrally diverse samples in PAGE also enabled evaluation of previously reported loci through comparison of effect sizes and explained phenotypic variances across diverse populations. The statistically significant attenuation of effect sizes in AA and HL populations was pervasive even after adjusting for the winner’s curse, with loci explaining a higher proportion of phenotypic variance in EA populations. The only exception was NEU, with the Duffy/*DARC* null variant making a substantial contribution to the variance in AA populations, which is consistent with previous findings. Accurate estimation of variant effects on the associated trait is crucial for risk prediction based on polygenic risk scores (PRS), and extra caution should be taken when using European-derived effect estimates in other ancestral groups.

Compared to the PAGE global paper [28], the current analyses included more samples and evaluated more phenotypes. We included samples genotyped on the MEGA array as well as additional samples genotyped on other Illumina or Affymetrix arrays from the participating studies, leading to more than a 128% increase in sample size compared to the PAGE global paper. In addition, the current analyses included eight phenotypes while the global paper focused on WBC and PLT. Our study has several limitations. First, the sample sizes of the underrepresented AA and HL populations remained limited compared to sample sizes available in Euro-centric GWAS (with over 500,000 EA participants in the BCX Consortium [23]). The relatively modest sample sizes limited the power to identify additional novel loci in the univariate association analyses and the multi-trait association analysis. Second, we were unable to examine the underrepresented Native American and Hawaiian populations. These participants were included in our PAGE Study but had limited numbers of white blood cell and platelet trait measurements. Studies on these ancestral groups currently are extremely sparse and continued efforts to include them in genetic association analyses are needed. Third, the usage of the European reference transcriptome may have introduced bias and the relatively limited sample sizes may have contributed to the absence of novel gene findings in the PrediXcan analysis, reinforcing the need to collect transcriptomics data and construct tailored models in minority populations.

In conclusion, the ancestrally diverse populations in the PAGE Study facilitated the discovery of both ancestry-specific and ancestry-agnostic findings at putative novel loci and previously established regions for association with white blood cell and platelet traits. Successful replication of multiple previously reported loci in PAGE indicated considerable shared genetic architecture underlying these traits. Our results emphasize the importance of improving diversity and inclusion in genetic association studies by incorporating participants with diverse ancestral backgrounds.

## Conclusions

We identified six potential novel findings for five of the eight examined white blood cell and platelet traits in the ancestrally diverse populations from PAGE. Multiple established loci were confirmed in our analysis and independent signals were identified in six reported regions. Systematic evaluation of white blood cell and platelet traits associated loci from a Euro-centric GWAS showed global attenuation of effect sizes in AA and HL compared to EA populations. Our results indicated the importance of diversity and inclusion in genetic association studies, which will lead to an improved understanding of these complex traits.

## Methods

### Study populations

In the discovery stage, our analysis included up to 64,784 participants of self-identified AA (n=16,201), HL (n=21,347), or EA (n=27,236) race/ethnicity from four cohort studies and one biobank (Table 1): the Atherosclerosis Risk in Communities Study (ARIC), the Coronary Artery Risk Development in Young Adults Study (CARDIA), the Hispanic Community Health Study/Study of Latinos (HCHS/SOL), the Women’s Health Initiative (WHI), and the BioMe™ Biobank (BioMe) (Supplemental Table 1). All participants provided informed consent and each study was approved by the Institutional Review Board (Supplemental Methods).

### Phenotype measurement and quality control

We studied eight hematological traits as defined in the standard clinical complete blood count (CBC) analysis, measuring properties of white blood cells (WBC, BAS, EOS, LYM, MON, and NEU) and platelets (PLT and MPV). Counts of white blood cells and the five subtype cells as well as platelets were measured using automated hematology cell counters and following standardized laboratory protocols from blood draws at the earliest available visit. Each count was reported in trillions of cells per liter (10^9^/L).

QC of the measured traits were performed before analysis (Supplemental Table 1). When available, participants were excluded if they had ever been diagnosed with HIV or leukemia, were currently pregnant or receiving chemotherapy, or had a severe hereditary anemia (primarily sickle-cell disease, determined by genotype) at time of blood draw. To remove sources of technical and non-genetic biological variation, and thus increase our power to detect genetic associations, we removed outliers exceeding four standard deviations from the mean of each trait in the overall study population. Due to the small proportion of basophils and eosinophils in whole blood, the counts for these two traits are often below the detection limit and were then recorded as zero. Therefore, we randomly imputed a phenotype value from a uniform distribution ranging from 0 to a study-specific lower detection limit (ranging from 0.0067 in the HCHS/SOL to 0.1 in BioMe) for those with a complete blood count measurement that was below the detection limit and used this value in analysis. The assignment of low, but non-zero counts, allows these subjects to be included in the analysis, as it is known that these values are in fact (very) low.

### Genotyping, imputation and quality control

Among all included participants, 8831 AA and 19,484 HL participants were genotyped on the MEGA arra y[29], yielding a total of 1,705,969 genetic variants. Standard QC filters were applied at the individual level as well as the variant level (Supplemental Methods). After QC, variants were further imputed to 1000 Genomes Phase 3 data using SHAPEIT2 and IMPUTE (version 2.3.2), resulting in 39,723,562 imputed SNPs with IMPUTE info score ≥0.4. An additional 36,469 participants (7370 AA, 1863 HL, and 27,236 EA participants) previously genotyped using other Illumina or Affymetrix arrays were also included in the analysis, again using standard QC procedures (Supplemental Methods). Variants that passed QC were imputed to the 1000 Genomes Phase 3 reference panel in each study. We further excluded variants on a study-specific basis which had poor imputation quality (info score< 0.4) or an effective sample size < 35 (calculated as 2×MAF×(1-MAF)×N×info score, where MAF is minor allele frequency and N is sample size). In the ancestry-combined, AA-specific, HL-specific, and EA-specific samples, 61, 53, 57, and 64% of variants had allele frequencies below 1%, respectively.

### Statistical analysis

In the discovery stage, we performed both univariate GWAS analysis for each of the eight traits and aSPU simulation-based method which jointly tested all eight traits [40]. For WBC and the five subtypes (BAS, EOS, LYM, MON, and NEU), values were log10 transformed before association analysis. For PLT and MPV, raw values were used. For samples genotyped on the MEGA array, residual values for each trait were calculated from linear regression models after adjustment for age, age^2^, sex (when applicable), center (when applicable), and the first 10 principal components (PC). For samples previously genotyped on either other Illumina or Affymetrix arrays, residual values for each trait were calculated from linear regression models after adjustment for age, age^2^, sex (when applicable), center (when applicable), and the first 10 PCs calculated from an LD-pruned set of genotypes in each individual study. In the univariate GWAS analysis, we tested the association of each genetic variant with the rank-based inverse-normally transformed residual values in MEGA samples and in each individual study, respectively. All MEGA samples were pooled together for testing while association testing was performed by study and ancestral group in non-MEGA samples. These association analyses were performed using SUGEN, which is based on generalized estimating equations (GEE) allowing correlated errors for first or second-degree relatives and independent error distributions by self-reported race/ethnic group [41]. Association results from these studies were then combined through fixed-effect inverse-variance-weighted meta-analysis in METAL for each trait [42]. Both ancestry-combined and ancestry-specific meta-analyses were performed. Complete summary level results are available through dbGaP (phs000356).

To identify additional novel loci and evaluate evidence for shared genetic effects across all eight traits, we combined the trans-ethnic meta-analysis results from each univariate trait analysis using aSPU to generate a joint *P* value for each variant [40]. The aSPU approach uses a simulated reference distribution (based on Monte Carlo simulations [40] ) to evaluate whether the most powerful combination of univariate summary z-scores implies an association between each variant and one or more of the tested traits. In comparison with other available multi-trait methods, we chose aSPU because it exhibited low type 1 error rate in simulations, accommodated direction of effect, and showed computational efficiency enabling the test of millions of variants. We implemented aSPU using Julia 1.0 to optimize efficiency (https://github.com/kaskarn/aspu_julia).

Genome-wide and suggestive significant cutoffs were set as *P*<2E-9 and *P*<5E-8, respectively [28, 30]. Using guidelines for frequency-based thresholds [30] we set genome-wide significance at 2E-9 as new discovery in this study was likely to be rare/low-frequency. We additionally used a suggestive cutoff of 5E-8 for relatively common variants with MAF> 5%. Novel loci were defined as those that: (1) reached the genome-wide or suggestive significance threshold; (2) were located more than 1 Mb away from any reported variants associated with any of the eight traits; (3) were available in the pooled MEGA result or were available in at least two non-MEGA studies when not available in the pooled MEGA result. Novel associations were defined as those that: (1) reached the genome-wide or suggestive significance threshold; (2) were located more than 1 Mb away from any reported variants associated with the examined trait but located within 1 Mb from variants previously reported for any of the other hematological traits; (3) were available in the pooled MEGA result or were available in at least two non-MEGA studies when not available in the pooled MEGA result. All novel loci and novel associations exhibiting genome-wide or suggestive significance were moved forward to the replication stage. These novel findings were examined in the publicly available summary statistics from the BCX (http://www.mhi-humangenetics.org/en/resources). There are two studies, BioMe and WHI, that were included in our discovery analysis and the BCX results as well. The fixed effect meta-analysis provided by BCX is a combination of the results of overlapping samples in WHI and BioMe and other remaining samples in BCX. As we know the association results for the overlapping samples in WHI and BioMe we can “invert” the fixed effect meta-analysis reported on the BCX website to obtain the results of all non-overlapping samples in BCX.

To identify distinct association signals at previously reported loci, we performed stepwise conditional analysis in each study, followed by meta-analysis. At each known locus that reached genome-wide significance in the ancestry-combined meta-analysis (*P*<2E-9), we identified the lead variant with the lowest *P* value and defined a 2 Mb region centered on the lead variant. At the *DARC* locus, the examined region was extended to ~ 6.5 Mb due to the extensive LD. We included the genotype dosage for the lead variant in each region as an additional covariate in the regression model. We did not stop the conditional analysis until all variants in each region showed *P*>2E-9.

Phenotypic variance explained by each genetic variant was calculated using the equation [43]

Explained phenotypic variance = $\frac{2{\beta}^2 MAF\left(1- MAF\right)}{2{\beta}^2 MAF\left(1- MAF\right)+S{E}^2\ 2N\  MAF\left(1- MAF\right)}$,

where β denotes the effect size of the variant on the associated trait and SE denotes standard error of the effect size.

### PrediXcan analysis

PrediXcan is a multi-omics approach for identifying genes associated with a trait of interest [44], which uses a reference database of derived genotype weights to impute unobserved gene expression levels into a set of genotyped samples. Gamazon et al. provided imputation models for gene expression in 48 different human tissues with Genotype-Tissue Expression (GTEx) V7 data, using elastic net regression with all cis-variants (defined as within 1 Mb of the gene) with MAF> 5% [44]. We performed a PrediXcan analysis to identify associations of WBC and PLT levels with these imputed values, representing GREx [44], in five disease-relevant tissues and cell types: whole blood, liver, spleen, thyroid, and Epstein-Barr virus (EBV) transformed lymphocytes. First, GREx in over 28,518 PAGE minority ancestry participants (AA, HL, Asian, and Hawaiian ancestry) genotyped on the MEGA array were imputed. Associations between the GREx and the traits (which were only available for WBC and PLT in AA and HL populations) were then estimated using SUGEN, both in an ancestry-combined and ancestry-specific manner. Genes with FDR < 0.05 in the ancestry-combined analysis were considered significant. Novel genes were defined as those exhibiting FDR < 0.05 and located more than 1 Mb away from any reported variants.

### Functional annotation

Functional annotation of the novel findings listed in Table 2 was performed using a comprehensive annotation database constructed from the WGSA [45], gene-centric function (GTEx) and genome-wide functional prediction scores (DANN and Eigen PC)] and a custom UCSC analysis hub visualizing various important regions [enhancer and repressor activities, DNase I hypersensitive sites (DHS) and transcribed regions], which facilitated the prioritization of potential functional genes and variants. Variants with DANN rank score ≥ 0.9 were coded as deleterious [46], and variants with Eigen PC phred score ≥ 17 were coded as functional [47]. Independent signals identified in the stepwise conditional analysis were also examined using this functional database. Custom UCSC bed tracks included the most significant variant of each novel finding and the proxy variants that are in LD (r^2^≥0.4) with the most significant variant within ±1 Mb region. The LD proxies of the six novel findings were generated using either ancestry-specific or ancestry-combined data (sample size-weighted LD using MEGA AA and HL samples for *INSIG1*, *MED13L*, and *HADHB*, sample size-weighted LD using MEGA AA and HL samples plus EA samples from WHI and ARIC for *TG* and *IGF1*, and European-specific LD using EA samples from WHI and ARIC for *PPP1R16B*). Primary mononuclear cells, monocytes, neutrophils, natural killer cells, T and B cells from peripheral blood and primary hemotapoietic stem cells, the seven most relevant cells to the eight studied white blood cell and platelet traits, were selected to examine chromatin immunoprecipitation-sequencing (ChiP-seq) signals associated with enhancers (H3K27ac and H3K4m1), repressors (H3K27me3), and transcribed regions (H3K36me3) [48].

In addition, we examined our novel findings and their LD proxies, as well as the independent signals at previously reported loci, in two comprehensive data sets combining chromatin accessibility data derived from ATAC-seq in hematopoesis-related cell types and GWAS results for various blood cell traits in the UKBB (EA participants only, https://molpath.shinyapps.io/ShinyHeme/) [34] and the BCX Consortium (ancestrally diverse populations) [23] . These two data sets included variants selected from the fine-mapping analyses (PP < 0.01 in UKBB and < 0.001 in BCX, respectively), which also showed enrichment based on chromatin accessibility of various hematopoietic populations using gchromVAR [34].

## Supplementary Information

**Additional file 1.**

**Additional file 2.**

## Data Availability

Individual level phenotype and genotype data are available through dbGAP (phs000356.v2.p1). Multi-ancestral GWAS results from the BCX Consortium: http://www.mhi-humangenetics.org/en/resources/ GWAS results and chromatin accessibility data in hematopoesis-related cell types on European ancestry participants from the UK Biobank: https://molpath.shinyapps.io/ShinyHeme/ Chromatin accessibility data in hematopoesis-related cell types on multi-ancestral participants from the BCX Consortium: https://molpath.shinyapps.io/ShinyHeme/

## Abbreviations

AA: African American
AITD: Autoimmune thyroid diseases
ARIC: Atherosclerosis Risk in Communities Study
BAS: Basophil
BioMe: BioMe™ Biobank
BCX: Blood Cell Consortium
CAGE: Consortium for the Architecture of Gene Expression
CARDIA: Coronary Artery Risk Development in Young Adults Study
CBC: Complete blood count
ChiP-seq: Chromatin immunoprecipitation-sequencing
DANN: deleterious annotation of genetic variants using neural networks
DGN: Depression Genes and Networks
DHS: DNase I hypersensitive sites
EA: European ancestry
EBV: Epstein-Barr virus
EOS: Eosinophil
eQTL: Expression Quantitative Trait Locus
FDR: False discovery rate
GEE: Generalized estimating equations
GREx: Genetically regulated gene expression
GTEx: Genotype-Tissue Expression
GWAS: Genome-wide association studies
HCHS/SOL: Hispanic Community Health Study/Study of Latinos
HL: Hispanic/Latino
LD: Linkage disequilibrium
LYM: Lymphocyte
MAF: Minor allele frequency
MEGA: Multiethnic genotyping array
MON: Monocyte
MPV: Mean platelet volume
NESDA/NTR: Netherlands Study of Depression and Anxiety/the Netherlands Twin Register
NEU: Neutrophil
PAGE: Population Architecture using Genomics and Epidemiology
PC: Principal component
PLT: Platelet
PP: Posterior probability
PRS: Polygenic risk scores
QC: Quality control
UKBB: UK Biobank
WBC: White blood cell count
WGSA: Whole genome sequence annotator
WHI: Women’s Health Initiative
