# Correction to: Multi-ethnic genome-wide association analyses of white blood cell and platelet traits in the Population Architecture using Genomics and Epidemiology (PAGE) Study

**DOI:** 10.1186/s12864-021-07919-1

**Published:** 2021-09-13

**Authors:** Yao Hu, Stephanie A. Bien, Katherine K. Nishimura, Jeffrey Haessler, Chani J. Hodonsky, Antoine R. Baldassari, Heather M. Highland, Zhe Wang, Michael Preuss, Colleen M. Sitlani, Genevieve L. Wojcik, Ran Tao, Mariaelisa Graff, Laura M. Huckins, Quan Sun, Ming-Huei Chen, Abdou Mousas, Paul L. Auer, Guillaume Lettre, the Blood Cell Consortium, Weihong Tang, Lihong Qi, Bharat Thyagarajan, Steve Buyske, Myriam Fornage, Lucia A. Hindorff, Yun Li, Danyu Lin, Alexander P. Reiner, Kari E. North, Ruth J. F. Loos, Laura M. Raffield, Ulrike Peters, Christy L. Avery, Charles Kooperberg

**Affiliations:** 1 Public Health Sciences Division, Fred Hutchinson Cancer Research Center, Seattle, WA USA; 2 Department of Epidemiology, Gillings School of Public Health, University of North Carolina at Chapel Hill, Chapel Hill, NC USA; 3 The Charles Bronfman Institute for Personalized Medicine, Icahn School of Medicine at Mount Sinai, New York, NY USA; 4 Cardiovascular Health Research Unit, University of Washington, Seattle, WA USA; 5 Stanford University School of Medicine, Stanford, CA USA; 6 Department of Biostatistics, Vanderbilt University Medical Center, Nashville, TN USA; 7 The Vanderbilt Genetics Institute, Division of Genetic Medicine, Vanderbilt University Medical Center, Nashville, TN USA; 8 Pamela Sklar Division of Psychiatric Genomics, Icahn School of Medicine at Mount Sinai, New York, NY USA; 9 Department of Biostatistics, Gillings School of Public Health, University of North Carolina at Chapel Hill, Chapel Hill, NC USA; 10 The Framingham Heart Study, National Heart, Lung and Blood Institute, Framingham, MA USA; 11 Population Sciences Branch, Division of Intramural Research, National Heart, Lung and Blood Institute, Framingham, MA USA; 12 Montreal Heart Institute, Montreal, Quebec Canada; 13 School of Public Health, University of Wisconsin–Milwaukee, Milwaukee, WI USA; 14 Department of Medicine, Faculty of Medicine, Université de Montréal, Montreal, Quebec Canada; 15 School of Public Health, University of Minnesota, Minneapolis, MN USA; 16 School of Medicine, University of California Davis, Davis, CA USA; 17 Medical School of University of Minnesota, Minneapolis, MN USA; 18 Department of Statistics and Biostatistics, Rutgers University, Piscataway, NJ USA; 19 Brown Foundation Institute for Molecular Medicine, the University of Texas Health Science Center, Houston, TX USA; 20 Division of Genomic Medicine, NIH National Human Genome Research Institute, Bethesda, MD USA; 21 Department of Genetics, Gillings School of Public Health, University of North Carolina at Chapel Hill, Chapel Hill, NC USA

**Correction to: BMC Genomics 22, 432 (2021)**

**https://doi.org/10.1186/s12864-021-07745-5**

Following publication of the original article [1], it was reported that a number of authors were missing from the authorship list. The following fourteen authors were mistakenly captured as members of the Blood Cell Consortium instead of full authors: Weihong Tang, Lihong Qi, Bharat Thyagarajan, Steve Buyske, Myriam Fornage, Lucia A Hindorff, Yun Li, Danyu Lin, Alexander P Reiner, Kari E North, Ruth J F Loos, Laura M Raffield, Ulrike Peters, Christy L Avery.

They have been added as full authors and the original article has been updated.
