# Supplementary material for: Multi-ethnic genome-wide association analyses of white blood cell and platelet traits in the Population Architecture using Genomics and Epidemiology (PAGE) study
Source: BMC Genomics. 2021 Jun 9;22:432. doi: 10.1186/s12864-021-07745-5 (PMC8191001; doi:10.1186/s12864-021-07745-5)
Supplement: Supplementary file 2 — Additional file 2. [file 12864_2021_7745_MOESM2_ESM.docx]

**Supplemental Figures**

**Fig. S1 Locuszoom plots of the six novel findings identified in the discovery stage.** Genetic coordinates are displayed along the x-axis (hg19) and genome-wide association significance level is plotted against the y-axis as -log10(*P-value*). LD with the lead variant at each locus was generated using MEGA AA samples, MEGA HL samples, and EA samples from ARIC and WHI through sample size-weighted approach. LD is indicated by color scale in relationship to the most significant variant (colored as purple diamond) in each association (red: r2≥0.8, orange: 0.6≤r2<0.8, green: 0.4≤r2<0.6, blue: 0.2≤r2<0.4, navy: r2<0.2). (A) *TG*; (B) *INSIG1*; (C) *IGF1*; (D) *MED13L*; (E) *HADHB*; (F) *PPP1R16B*.

(A)


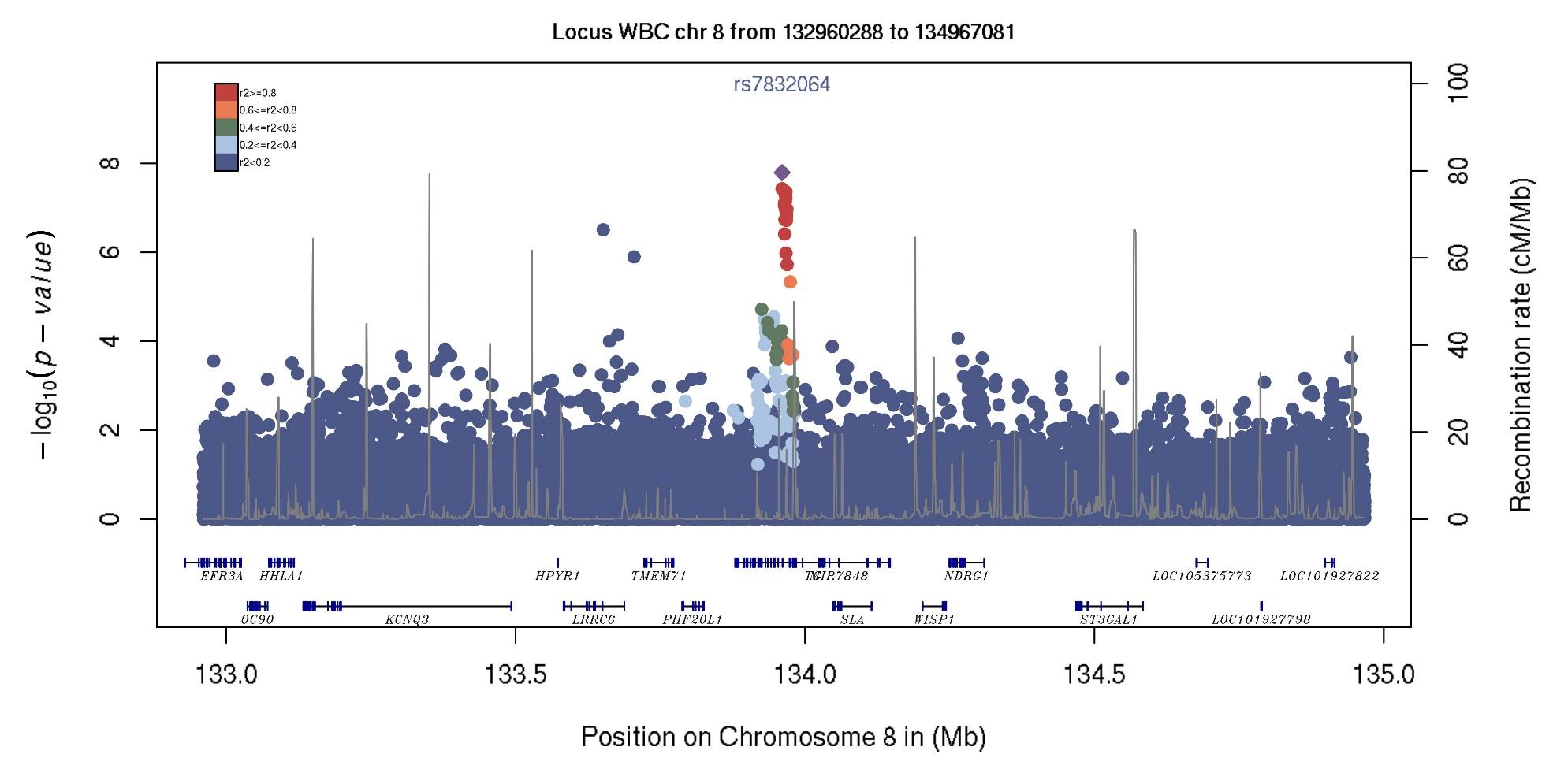


(B)


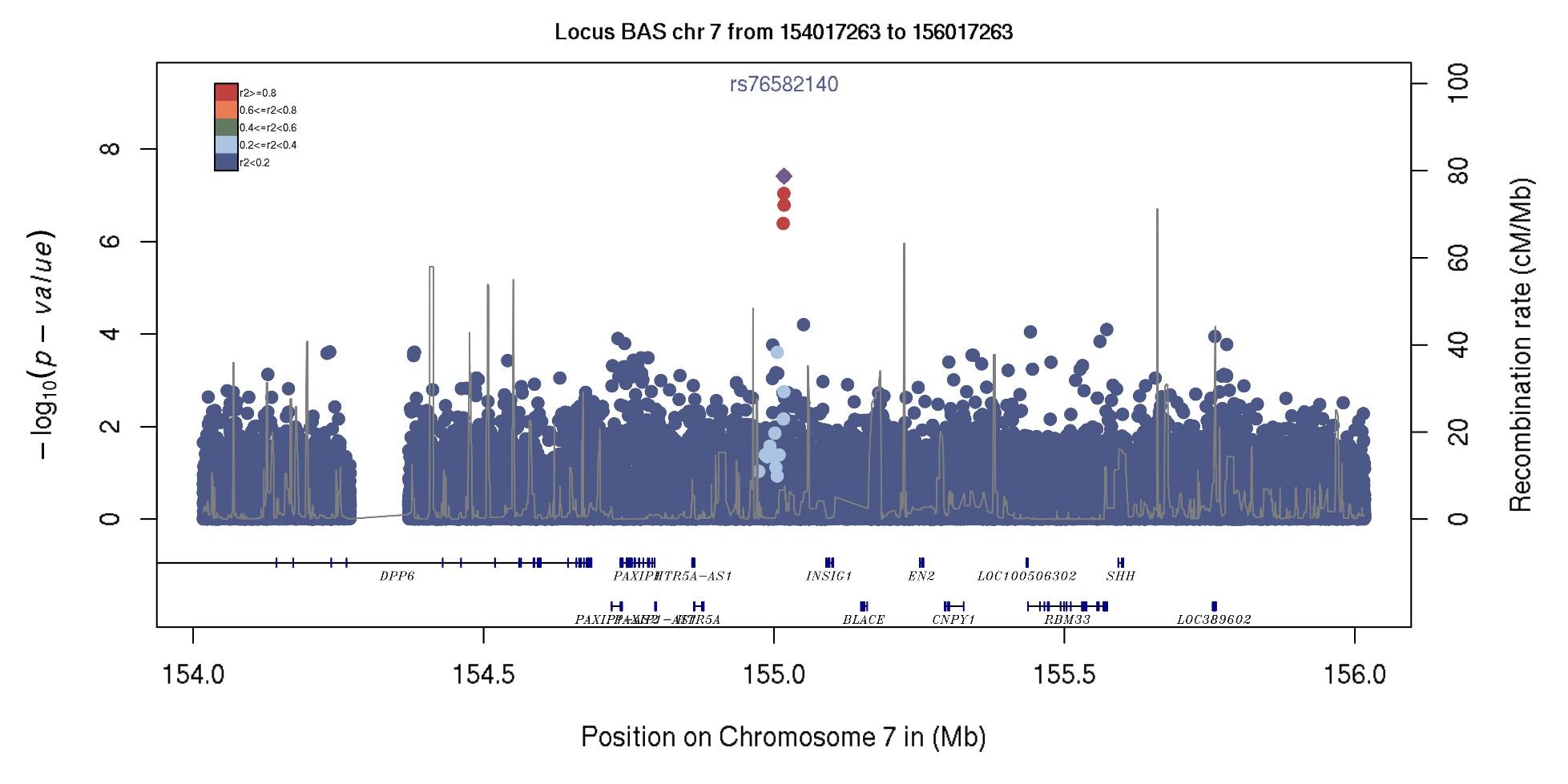


(C)


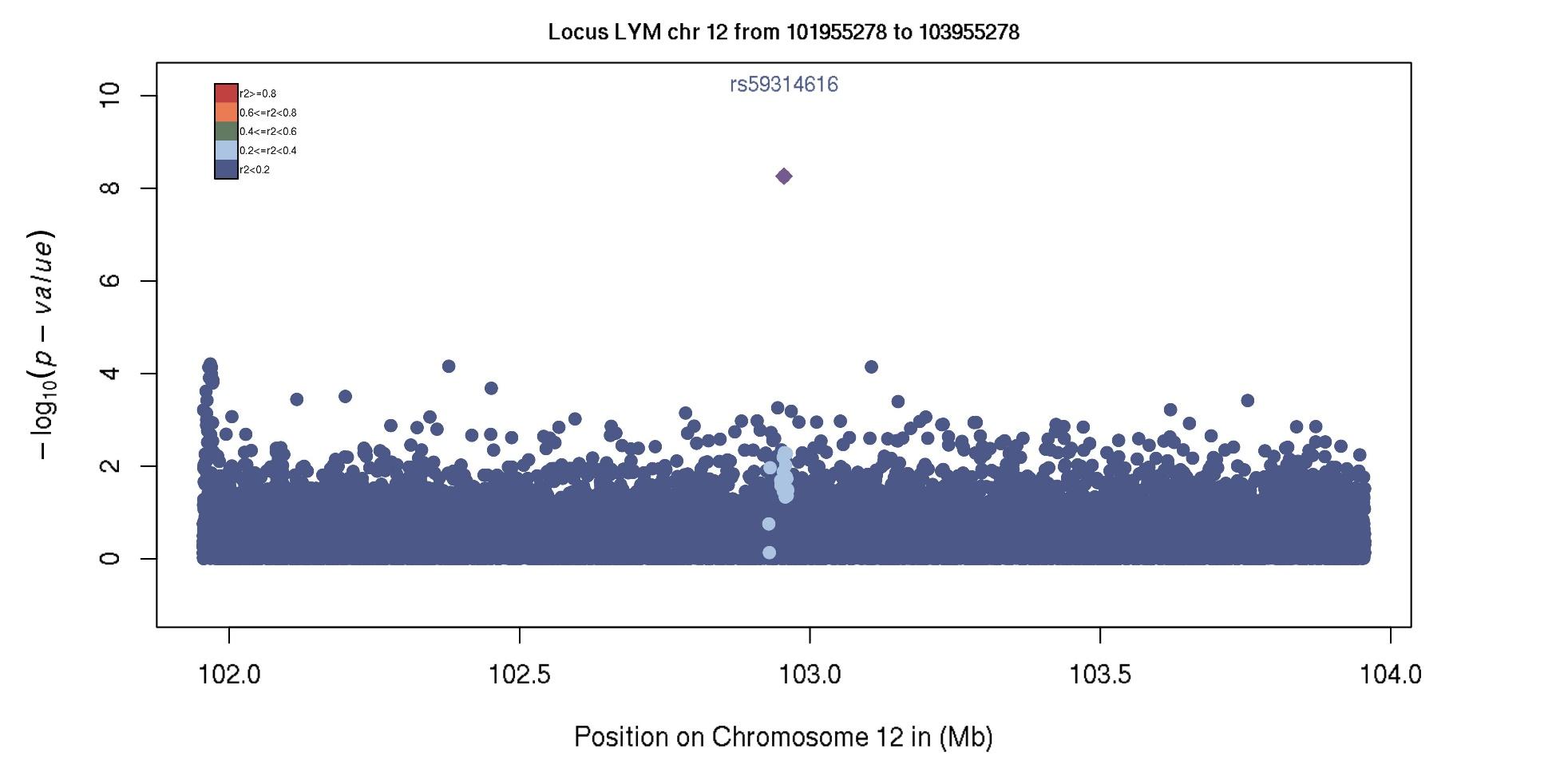


(D)


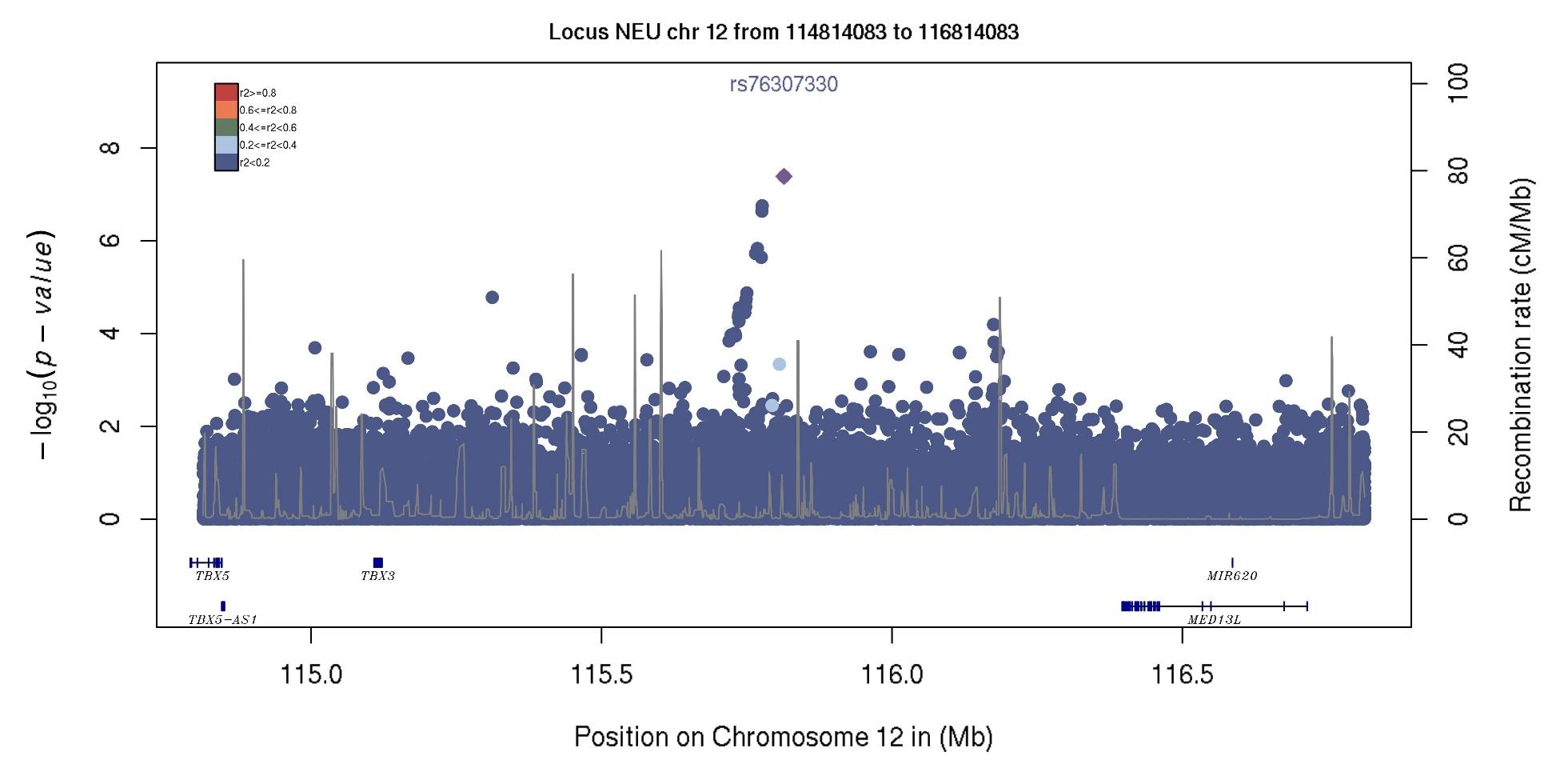


(E)


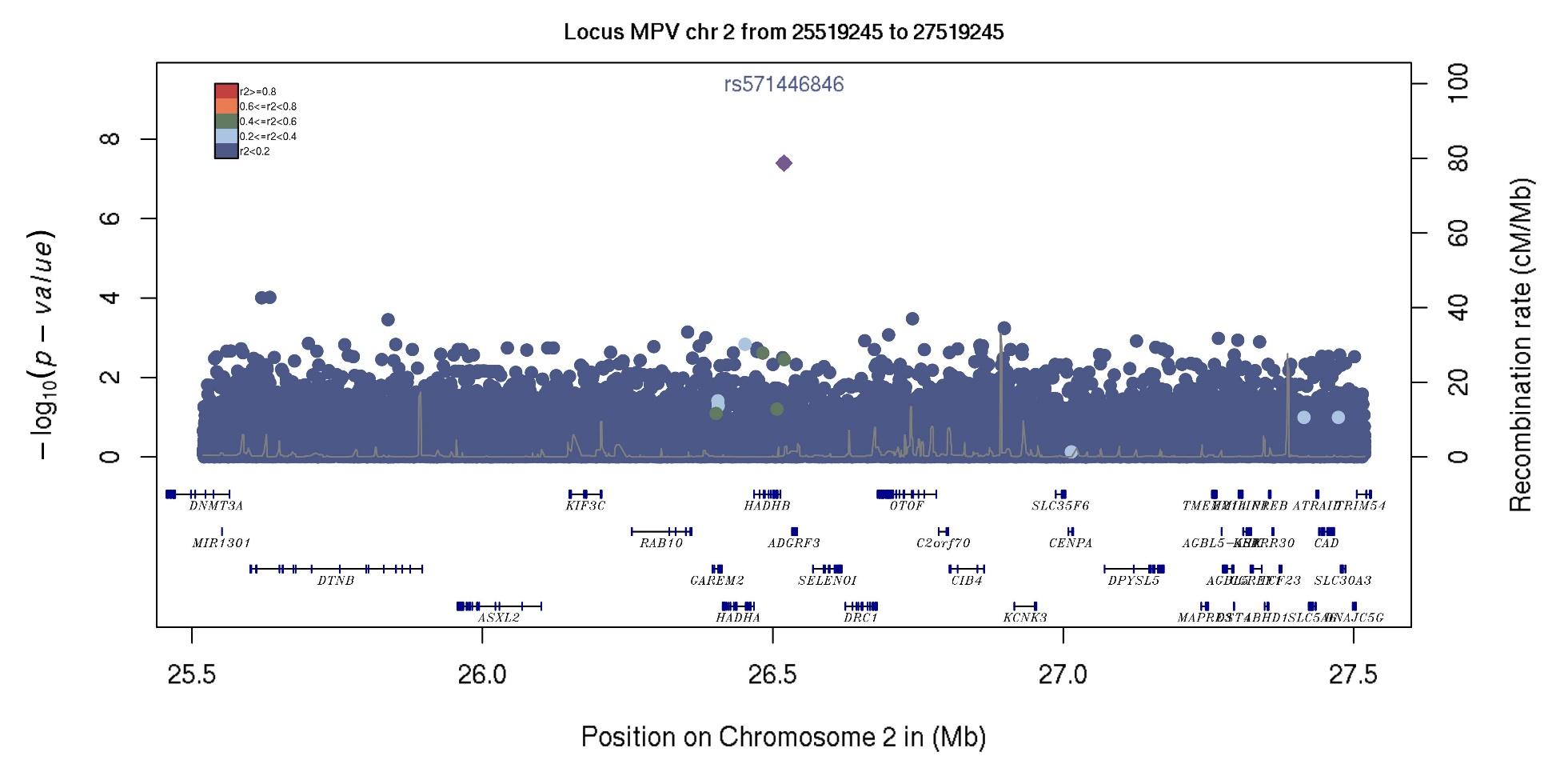


(F)


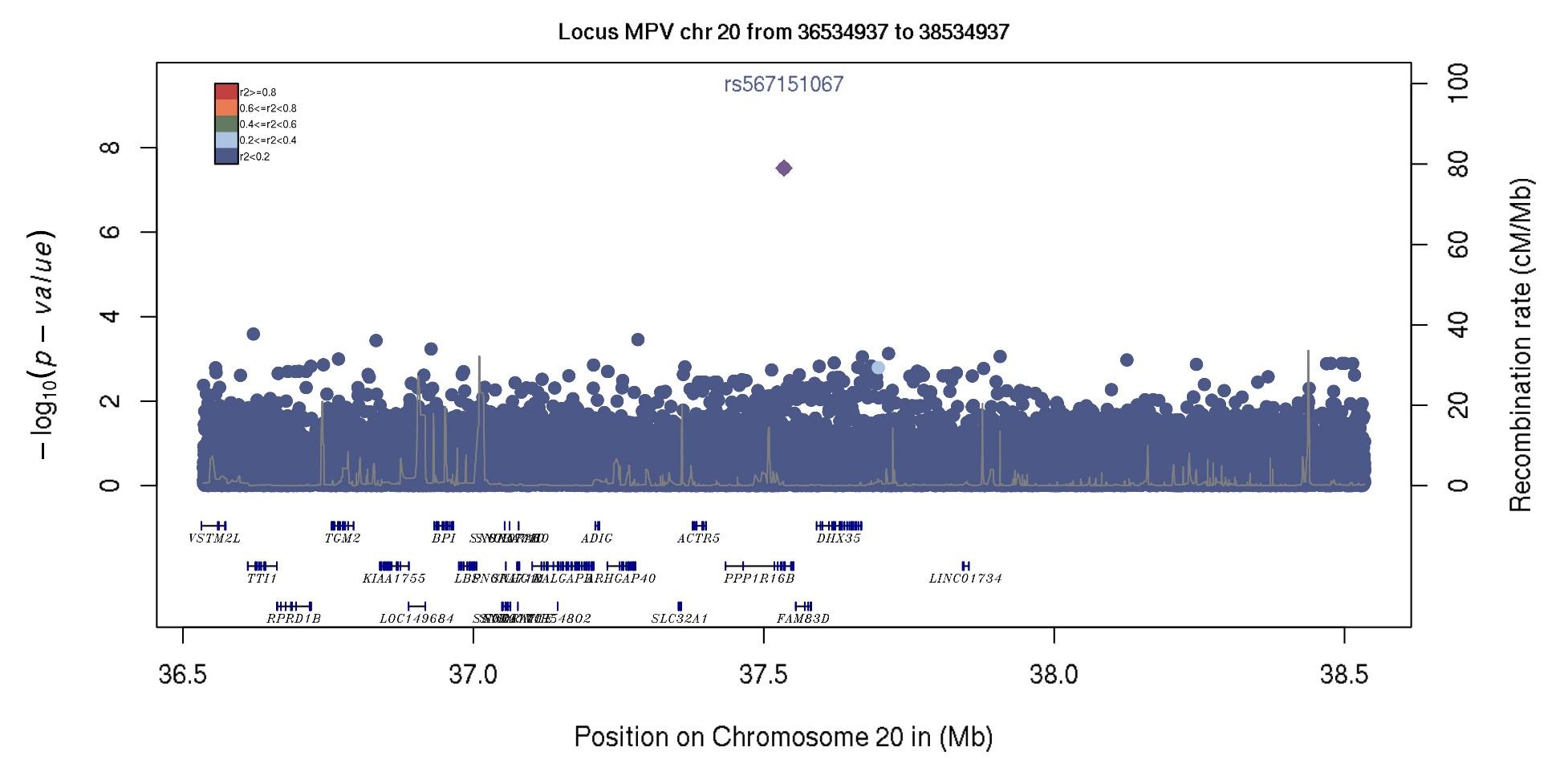


**Fig. S2 Comparison of effect estimates across AA, HL, and EA populations in PAGE.** All independent variants reported by Astle. et al for each trait that are available in all three ancestral groups in PAGE were included in the comparison. The black line are the identity lines. (A) WBC; (B) BAS; (C) EOS; (D) LYM; (E) MON; (F) NEU; (G) PLT; (H) MPV.

(A)

**
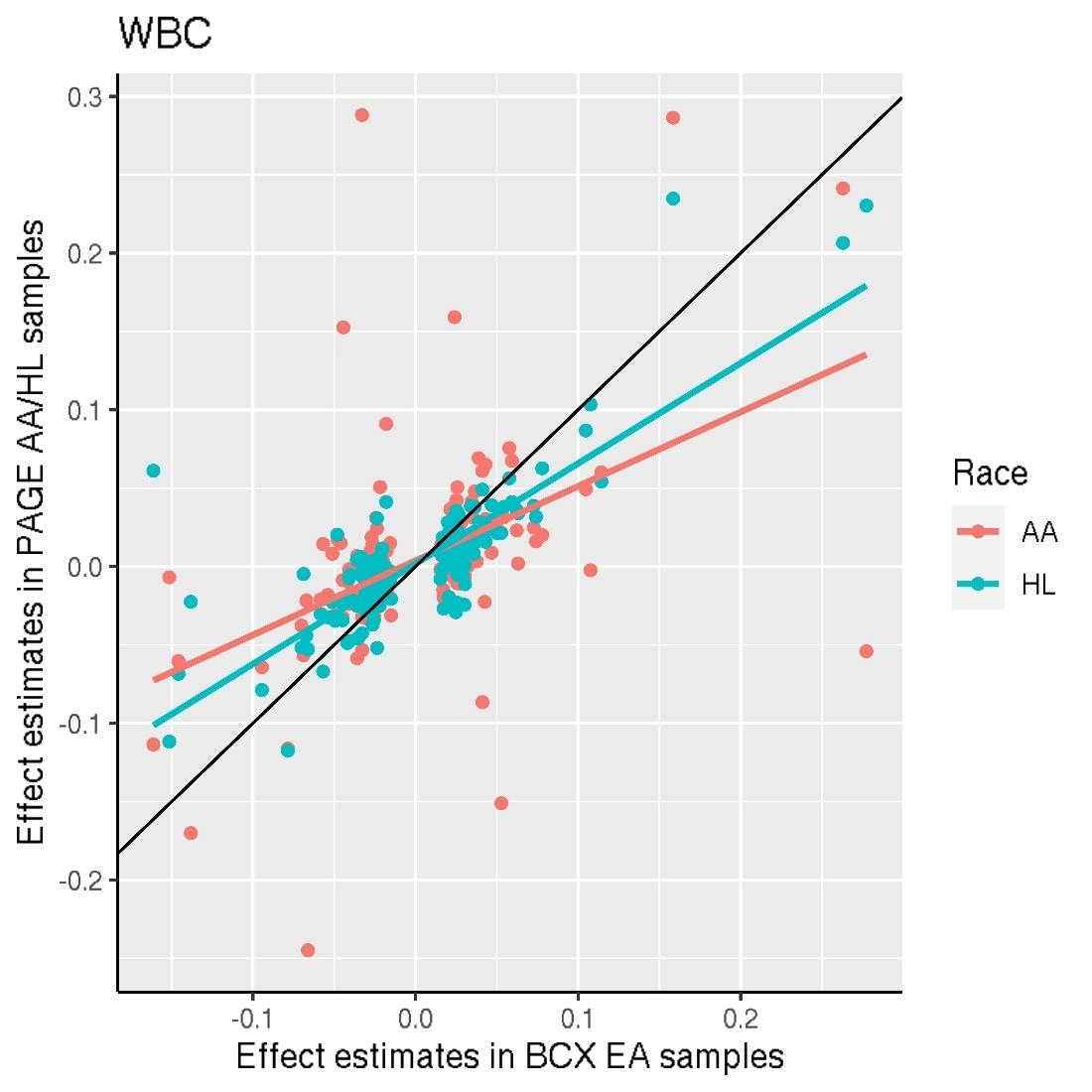
**

(B)

**
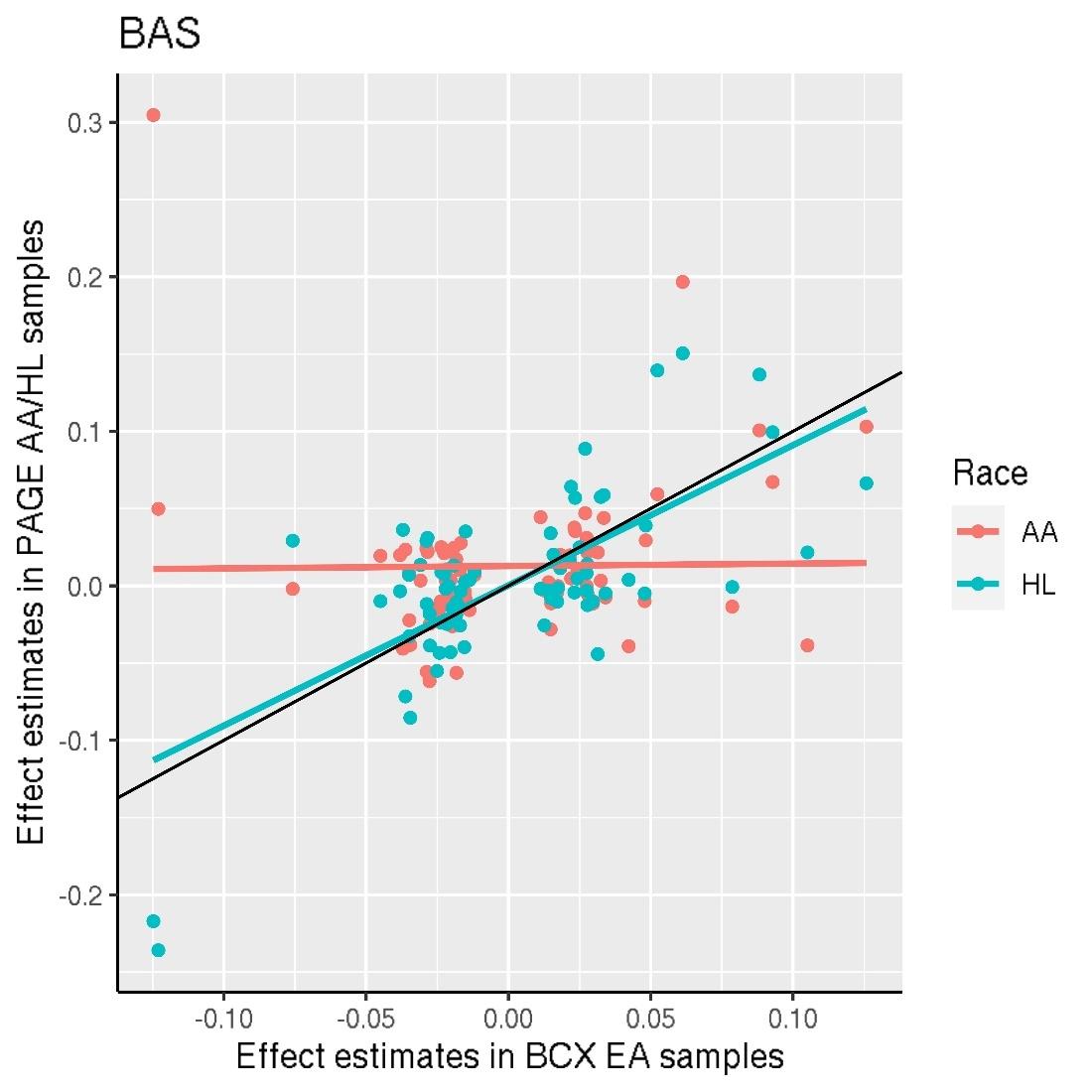
**

(C)

**
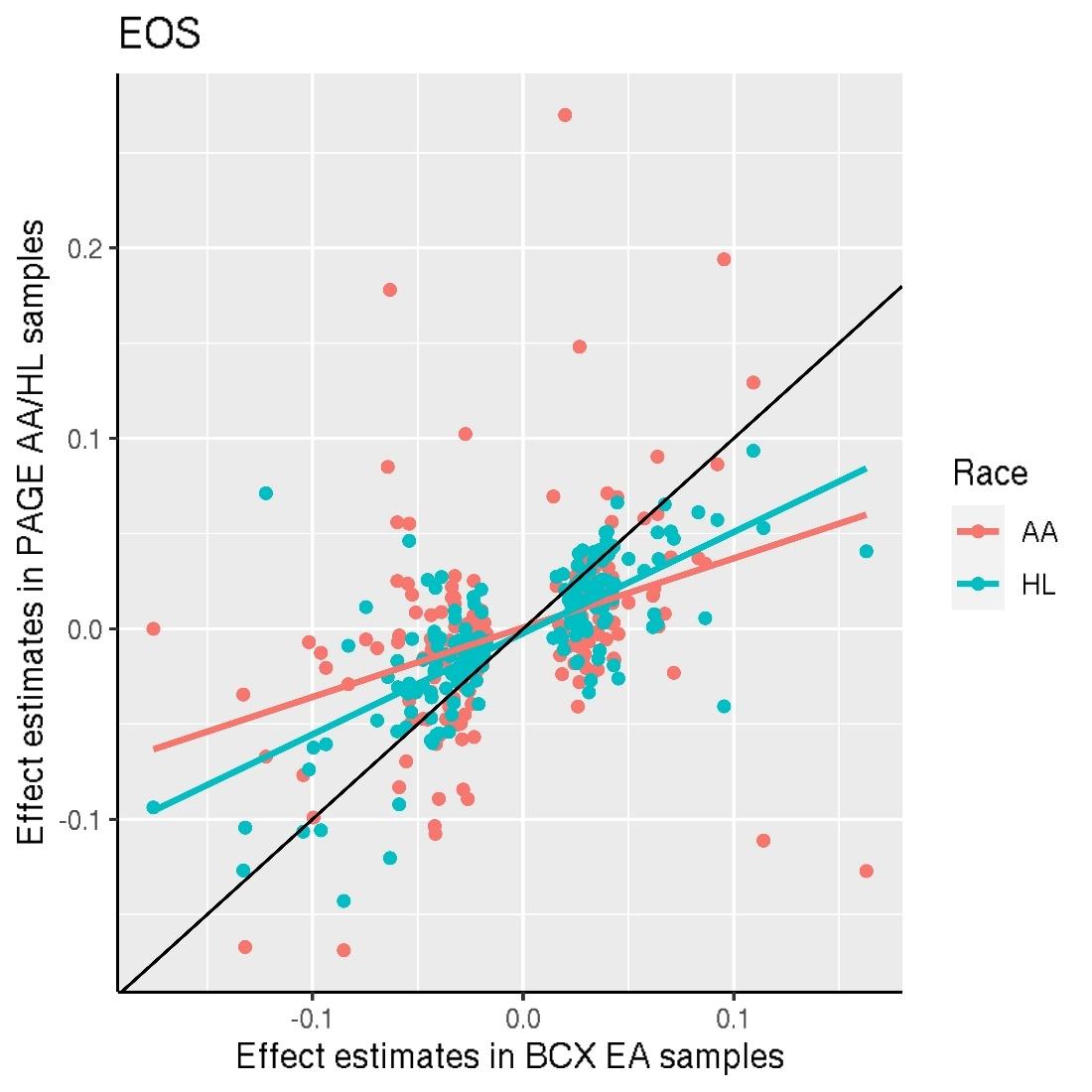
**

(D)

**
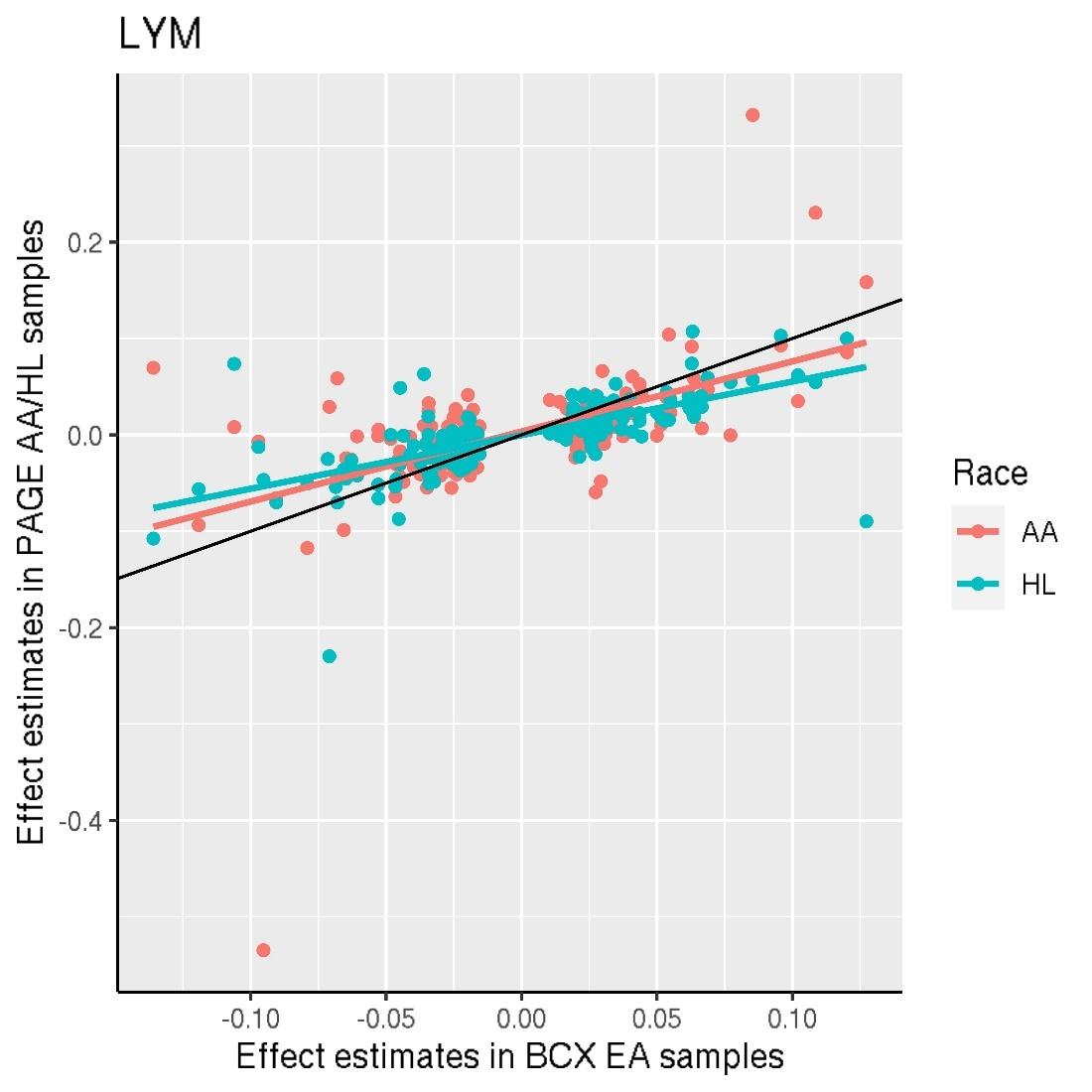
**

(E)

**
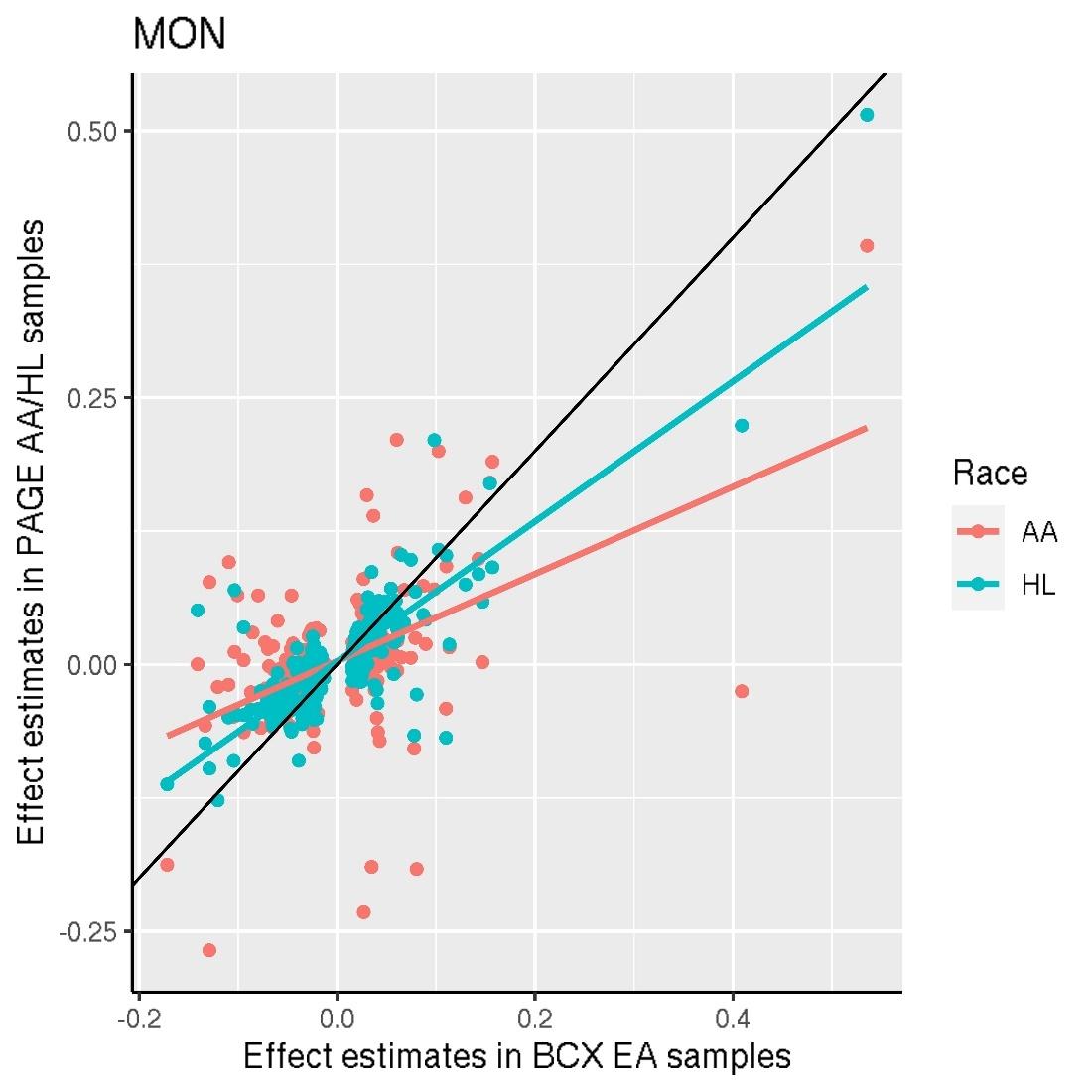
**

(F)

**
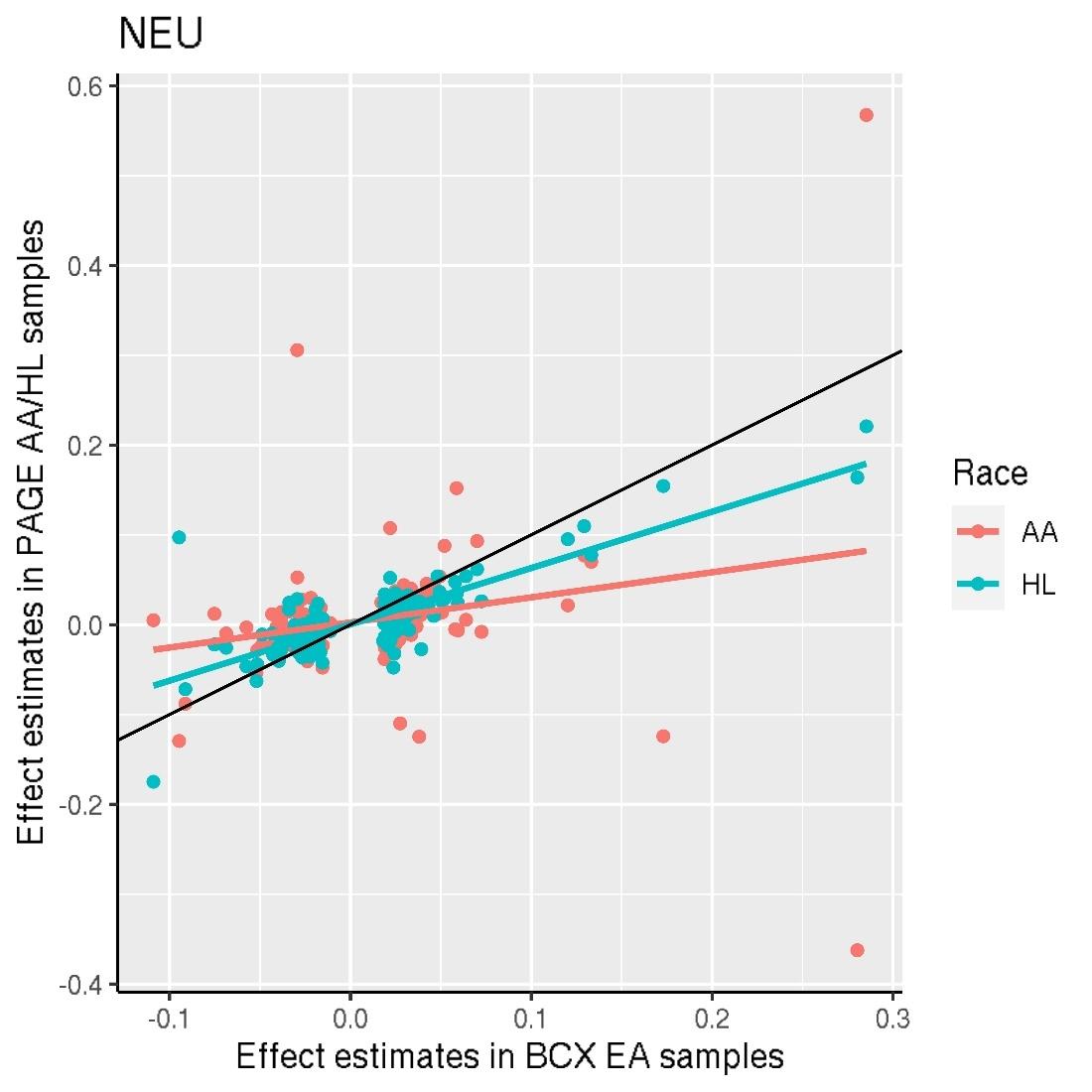
**

(G)

**
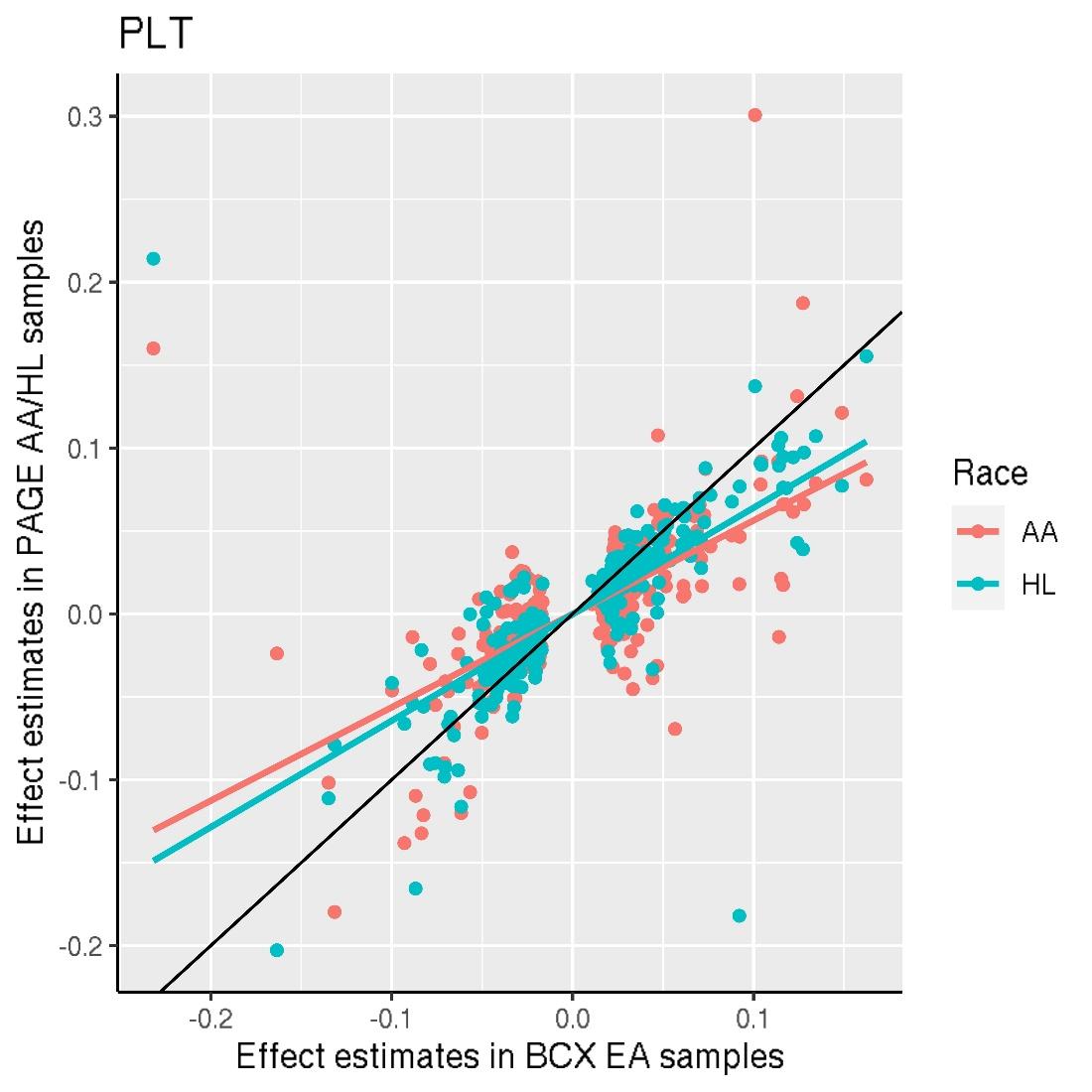
**

(H)

**
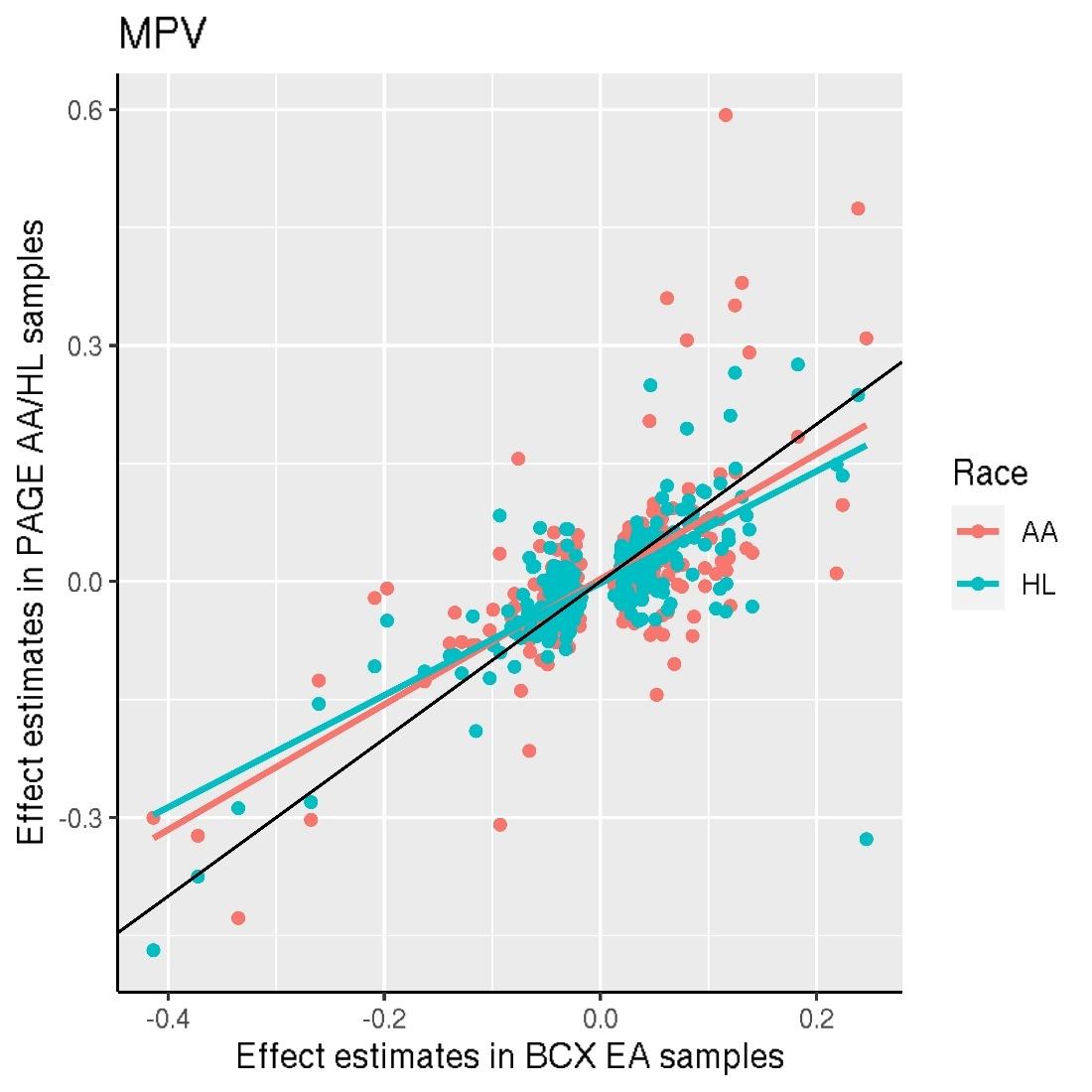
**

**Fig. S3 Functional annotation of the six novel findings in PAGE.** The lead variant at each locus was colored purple, and the LD proxies showing r^2^≥0.8, 0.8>r^2^≥0.6, and 0.6>r^2^≥0.4 were colored red, orange, and green, respectively. (A) *TG*; (B) *INSIG1*; (C) *IGF1*; (D) *MED13L*; (E) *HADHB*; (F) *PPP1R16B*.

(A)


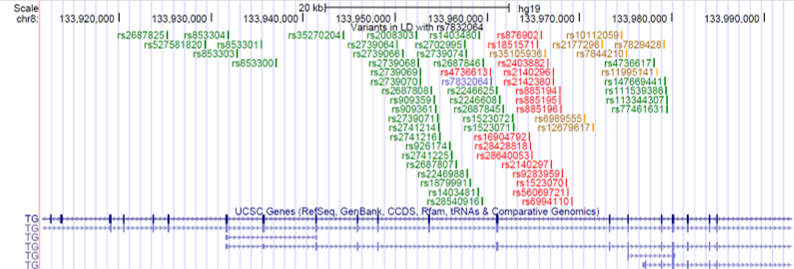


(B)


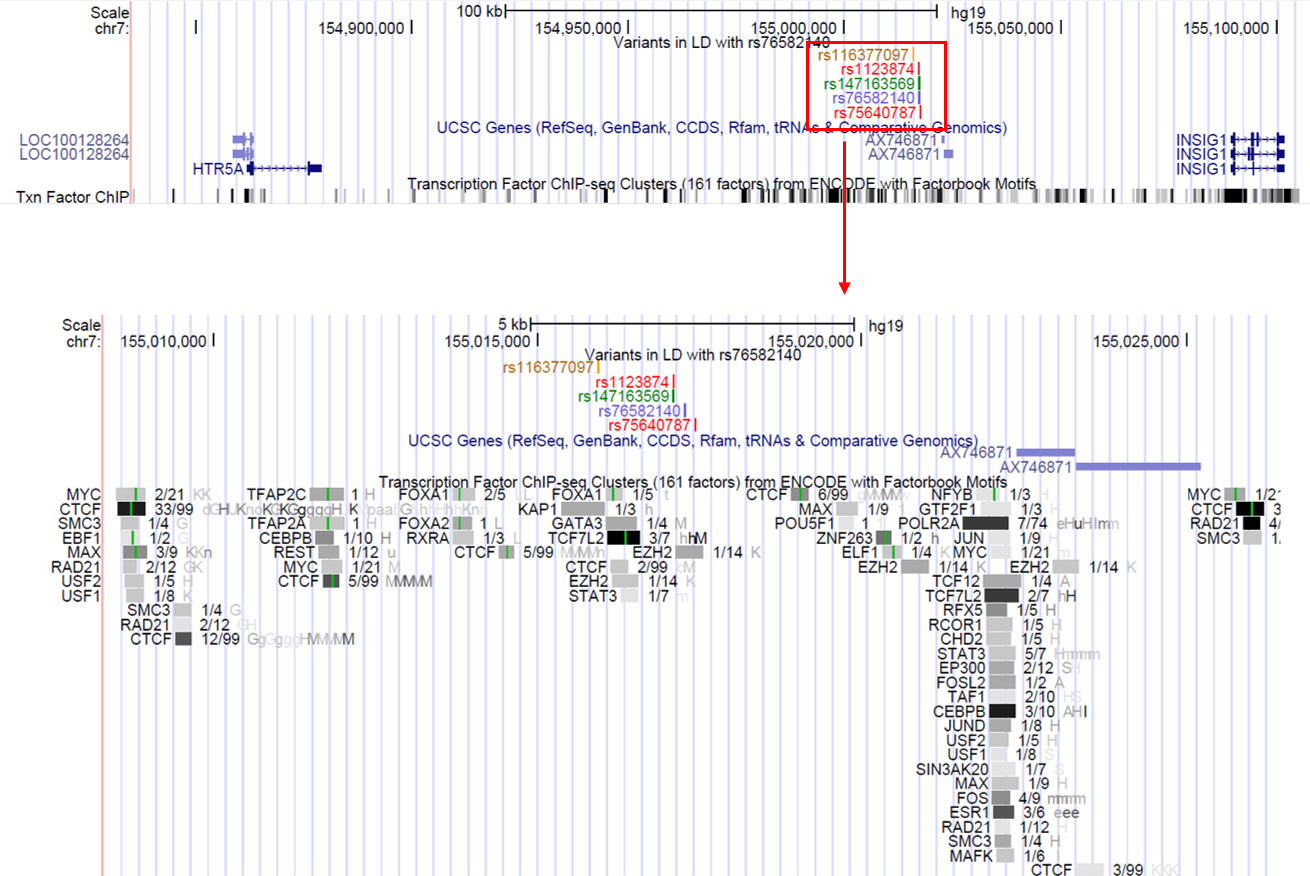


(C)


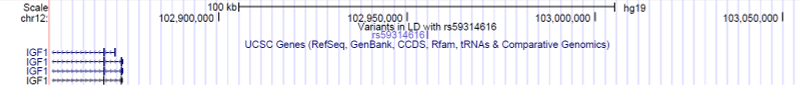


(D)


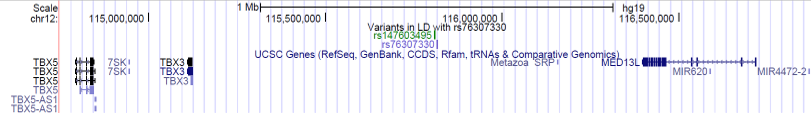


(E)


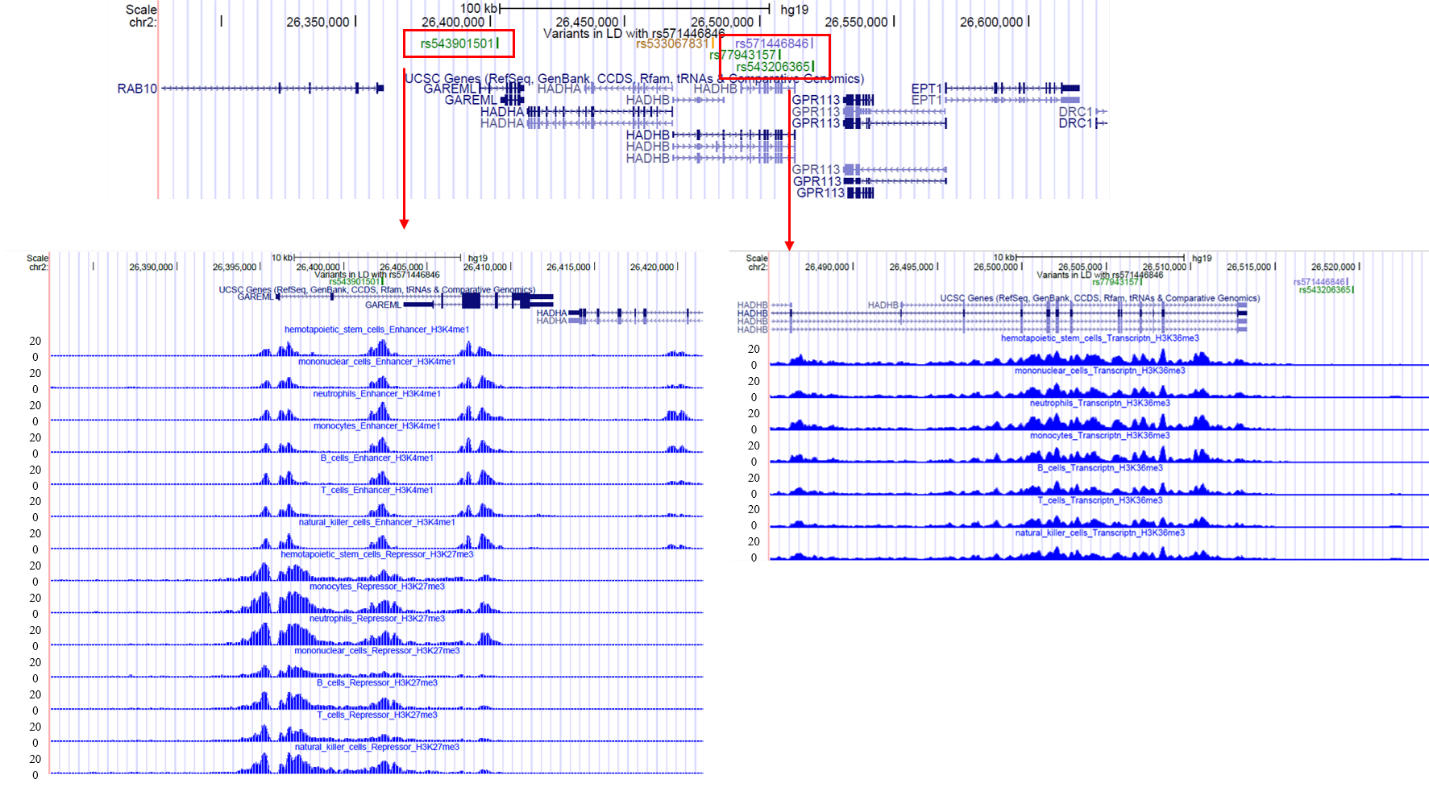


(F)


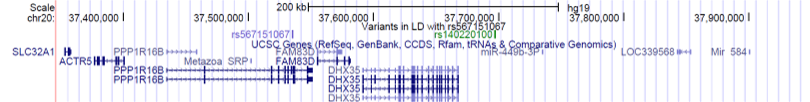


**Supplemental Methods**

1. Participating studies in PAGE

BioMe

The Charles Bronfman Institute for Personalized Medicine at Mount Sinai Medical Center (MSMC), BioMe^TM^ BioBank (BioMe) is an EMR-linked bio-repository drawing from Mount Sinai Medical Center consented patients which were drawn from a population of over 70,000 inpatients and 800,000 outpatients annually. The MSMC serves diverse local communities of upper Manhattan, including Central Harlem (86% African American), East Harlem (88% Hispanic/Latino), and Upper East Side (88% Caucasian/White) with broad health disparities. BioMe enrolled over 26,500 participants from September 2007 through August 2013, with 25% African American, 36% Hispanic/Latino (primarily of Caribbean origin), 30% Caucasian, and 9% of Other ancestry. The BioMe population reflects community-level disease burdens and health disparities with broad public health impact. Biobank operations are fully integrated in clinical care processes, including direct recruitment from clinical sites waiting areas and phlebotomy stations by dedicated Biobank recruiters independent of clinical care providers, prior to or following a clinician standard of care visit. Recruitment currently occurs at a broad spectrum of over 30 clinical care sites. Study participants of self-reported European ancestry were not included in this analysis. (dbGaP study accession number: phs000925).

HCHS/SOL

The Hispanic Community Health Study / Study of Latinos (HCHS/SOL) is a multi-center study of Hispanic/Latino populations with the goal of determining the role of acculturation in the prevalence and development of diseases, and to identify other traits that impact Hispanic/Latino health [1]. The study is sponsored by the National Heart, Lung, and Blood Institute (NHLBI) and other institutes, centers, and offices of the National Institutes of Health (NIH). Recruitment began in 2006 with a target population of 16,000 persons of Cuban, Puerto Rican, Dominican, Mexican or Central/South American origin. Household sampling was employed as part of the study design. Participants were recruited through four sites affiliated with San Diego State University, Northwestern University in Chicago, Albert Einstein College of Medicine in Bronx, New York, and the University of Miami. Researchers from seven academic centers provided scientific and logistical support. Study participants who were self-identified Hispanic/Latino and aged 18-74 years underwent extensive psycho-social and clinical assessments during 2008-2011. A re-examination of the HCHS/SOL cohort is conducted during 2015-2017. Annual telephone follow-up interviews are ongoing since study inception to determine health outcomes of interest (dbGaP study accession number: phs000555).

MEC

The Multiethnic Cohort (MEC) is a population-based prospective cohort study including approximately 215,000 men and women from Hawaii and California [2]. All participants were 45-75 years of age at baseline, and primarily of five ancestries: Japanese Americans, African Americans, European Americans, Hispanic/Latinos, and Native Hawaiians. MEC was funded by the National Cancer Institute in 1993 to examine lifestyle risk factors and genetic susceptibility to cancer. All eligible cohort members completed baseline and follow-up questionnaires. WBC and differential counts were measured in ethylenediaminetetraacetic acid (EDTA) whole blood obtained at the baseline examination using a Sysmex XE-2100 instrument (Sysmex America) at the University of Minnesota according to national and international standards and procedures. Individuals pregnant at the time of blood draw, those with>5% circulating blasts or immature cells, end-stage renal disease or any hematologic malignancy, and those undergoing chemotherapy for solid tumors were excluded from our analyses.

WHI

The Women’s Health Initiative (WHI) is one of the largest (n=161,808) studies of women’s health ever undertaken in the U.S. There are two major components of WHI: (1) a clinical trial (CT) that enrolled and randomized 68,132 women ages 50–79 into at least one of three placebo control clinical trials (hormone therapy, dietary modification, and supplementation with calcium and vitamin D); and (2) an observational study (OS) that enrolled 93,676 women of the same age range into a parallel prospective cohort study [3]. A diverse population including 26,045 (17%) women from minority groups was recruited from 1993–1998 at 40 clinical centers across the U.S. Details on the study design, eligibility, recruitment, and the reliability of the baseline measures of demographic and health characteristics have been published elsewhere [3,4]. Among the U.S. minority participants enrolled in WHI, 12,468 women (including 6,829 self-identified African American and 4,626 self-identified Hispanic subjects) consenting to genetic research were included in PAGE II for genotyping with the Multi-Ethnic Genotyping Array (MEGA) [5]. Fasting blood samples were obtained from all participants at baseline and were analyzed for WBC (and subtype) count and platelet count by certified laboratories at each of the 40 clinical centers as part of a complete blood count [4]. Results were entered into the WHI database at each clinical center and were reviewed by clinical center staff [6]. In year 3, a second blood sample was obtained from women in the observational study; data on blood parameters (including WBC count) measured at this visit were available for 81% of observational study participants (74,375/93,676). These assays were performed in a single laboratory using the same methods. Complete blood counts (CBC) were measured within 30 hours of draw. In addition to the main WHI CT and OS, six ancillary studies contributed existing GWAS data and CBC measurements to this study (SHARe, GARNET, LLS, WHIMS, GECCO, and HIPFX, Supplemental Table 1).

ARIC

The Atherosclerosis Risk in Communities Study (ARIC) is a prospective population-based study of atherosclerosis and cardiovascular diseases in 15,792 men and women, including 11,478 non-Hispanic whites and 4,314 African Americans drawn from four U.S. communities (suburban Minneapolis, Minnesota; Washington County, Maryland; Forsyth County, North Carolina; and Jackson, Mississippi) [7]. Participants were between ages 45 and 64 years at their baseline examination in 1987–1989, when blood was drawn for DNA extraction and participants consented to genetic testing. Blood for complete blood-count analysis was drawn at the baseline exam.

CARDIA

The Coronary Artery Risk Development in Young Adults (CARDIA) study is a prospective, multi-center investigation of the natural history and etiology of cardiovascular disease in a cohort of African Americans and whites who were 18–30 years of age at the time of initial examination. The CARDIA sample was recruited at random during 1985–1986 primarily from populations based in Birmingham, Alabama; Chicago, Illinois; and Minneapolis, Minnesota; and Oakland, California from the membership of the Kaiser-Permanente Health Plan. The initial examination included 5,115 participants selectively recruited to represent proportionate racial, gender, age, and education groups from each of the four communities. From the time of initiation of the study in 1985–1986 (baseline examination), six follow-up examinations were conducted 2, 5, 7, 10, 15, 20, and 25 years later. DNA extraction for genetic studies was performed at the 10-year examination. After the availability of adequate amounts of high-quality DNA was taken into account and appropriate informed consent and genotyping quality-control and assurance procedures were put in place, genome-wide genotype data were available for 955 African Americans, of whom 953 were included in the current analysis.

2. Genotyping, imputation, and quality control

Details on PAGE genotyping have previously been described [8]. In brief, PAGE samples were genotyped using the Multi-Ethnic Genotyping Array (MEGA) [5], which includes a GWAS scaffold designed to tag both common and low frequency variants in global populations. Additionally, it contains enhanced tagging in exonic regions, hand-curated content to interrogate clinically relevant variants, and enriched coverage to fine-map known GWAS loci. DNA was isolated from blood (HCHS/SOL and BioMe) and buffy coat (WHI). DNA samples were genotyped at the Center for Inherited Disease Research (CIDR) using the MEGA, and genotypes were called using the GenomeStudio version 2001.1, Genotyping Module 1.9.4, and GenTrain version 1.0. Preliminary quality control (QC) was performed at CIDR and included removal of samples showing sex/gender discrepancies, Mendelian inconsistencies, unexpected duplication, unexpected non-duplication, poor genotyping performance, or evidence of DNA mixture. Further Quality Assurance / Quality Control (QA/QC) was performed by the University of Washington Genetics Coordinating Center (UWGACC), and information from PAGE study investigators to remove samples with identity issues, restricted consent, and duplicate scans. A total of 1,705,969 variants were genotyped on the MEGA array. After variant-level QC, a total of 1,438,399 variants remained for the following analysis.

In PAGE, imputation was conducted by the UWGACC. Genotyped variants passing QA/QC were further restricted to sites with (1) known chromosome and position; (2) location on chromosomes 1-22, X, or XY (pseudo-autosomal); (3) with unique positions, which involved removing redundant and duplicate sites; and (4) sites with available strand annotation. After these restrictions, a total of 1,402,653 sites remained for imputation. The study samples were phased with SHAPEIT2 [9] and imputed with IMPUTE2 [10] to the 1000 Genomes Project Phase 3 data release [11]. Imputed variants were excluded from further analyses if the IMPUTE2 info score was less than 0.4. Segments of the genome which were known to harbor gross chromosomal anomalies were filtered out of the final genotype probabilities files. A total of 39,723,562 imputed variants passed QC.

In the sample-level QC, we removed a small number of samples that were supposed to be duplicates but had a concordance rate smaller than 90% as well as appeared duplicates but were from unrelated individuals. Additionally, as subjects for each GWAS were selected independently, we checked for duplicates between the studies. This resulted in a total of 64,784 unique subjects in the data with any trait measurement analyzed here.

**References**

1. Sorlie PD, Aviles-Santa LM, Wassertheil-Smoller S, et al. Design and implementation of the Hispanic Community Health Study/Study of Latinos. *Ann Epidemiol.* 2010;20(8):629-641.

2. Gram IT, Park SY, Kolonel LN, et al. Smoking and Risk of Breast Cancer in a Racially/Ethnically Diverse Population of Mainly Women Who Do Not Drink Alcohol. *American Journal of Epidemiology.* 2015;182(11):917-925.

3. Anderson GL, Manson J, Wallace R, et al. Implementation of the Women's Health Initiative study design. *Ann Epidemiol.* 2003;13(9 Suppl):S5-17.

4. Langer RD, White E, Lewis CE, Kotchen JM, Hendrix SL, Trevisan M. The Women's Health Initiative Observational Study: baseline characteristics of participants and reliability of baseline measures. *Ann Epidemiol.* 2003;13(9 Suppl):S107-121.

5. Bien SA, Wojcik GL, Zubair N, et al. Strategies for Enriching Variant Coverage in Candidate Disease Loci on a Multiethnic Genotyping Array. *Plos One.* 2016;11(12):e0167758.

6. Eaton CB, Young A, Allison MA, et al. Prospective association of vitamin D concentrations with mortality in postmenopausal women: results from the Women's Health Initiative (WHI). *Am J Clin Nutr.* 2011;94(6):1471-1478.

7. The Atherosclerosis Risk in Communities (ARIC) Study: design and objectives. The ARIC investigators. *Am J Epidemiol.* 1989;129(4):687-702.

8. Wojcik GL, Graff M, Nishimura KK, et al. Genetic analyses of diverse populations improves discovery for complex traits. *Nature.* 2019;570(7762):514-518.

9. Delaneau O, Marchini J, Zagury JF. A linear complexity phasing method for thousands of genomes. *Nat Methods.* 2011;9(2):179-181.

10. Howie BN, Donnelly P, Marchini J. A flexible and accurate genotype imputation method for the next generation of genome-wide association studies. *PLoS Genet.* 2009;5(6):e1000529.

11. Genomes Project C, Auton A, Brooks LD, et al. A global reference for human genetic variation. *Nature.* 2015;526(7571):68-74.
